# Intrinsic replication advantage of a Hepatitis B Virus genotype C strain is independent of the basal core promoter A1762T/G1764A mutation

**DOI:** 10.7717/peerj.21866

**Published:** 2026-11-05

**Authors:** Yihan Xiao, Xinjun Chen, Zongxin Li, Sheng Lei, Zhenbin Chen, Junqi Cai, Xiuji Cui

**Affiliations:** Key Laboratory of Tropical Translational Medicine of Ministry of Education, Hainan Medical University-The University of Hong Kong Joint Laboratory of Tropical Infectious Diseases, College of Basic Medical Sciences, The Second Affiliated Hospital of Hainan Medical University, Hainan Medical University, Haikou, China

**Keywords:** Hepatitis b virus, Basal core promoter, Chronic hepatitis b, Hainan

## Abstract

This study aimed to isolate and characterize individual hepatitis B virus (HBV) strains from a patient co-infected with genotype B and C, and to directly compare their intrinsic replication capacities. HBV DNA was isolated from a chronic hepatitis B patient. Genotypes were determined by genotype-specific polymerase chain reaction (PCR), and full-length genomes were cloned. Two clones representing subgenotype B2 (c17) and C1 (c21) were selected, sequenced and transfected into HepG2 cells. Viral replication was assessed *via* Southern blot and quantitative PCR. The role of the common Adenine (A)1762Thymine (T/)Guanine (G)1764A mutation in the C1 clone was investigated by site-directed mutagenesis. As a result, the genotype C1 clone exhibited significantly higher replication capacity than the B2 clone. Counterintuitively, reverting the A1762T/G1764A mutation in the C1 clone to wild-type further enhanced its replication, indicating this mutation was not the primary driver of its high replicative fitness. In conclusion, HBV genotype C1 possesses a greater inherent replication capacity than B2, independent of the classic basal core promoter (BCP) mutation, providing a virological explanation for its association with severe disease outcomes in co-infection.

## Introduction

Chronic Hepatitis B (CHB) remains a significant global health burden, with an estimated 4% of the Chinese population being carriers of the Hepatitis B virus (HBV) (Hui et al., 2024). This high prevalence is a major contributor to severe liver diseases, including liver cirrhosis and hepatocellular carcinoma (HCC), posing a substantial challenge to public health systems (Kao, 2003; Liu et al., 2023; Wang et al., 2014; Yuen & Lai, 2000).

HBV, a member of the *Hepadnaviridae* family, possesses a small (approximately 3.2 kb), relaxed circular DNA genome (Delius et al., 1983). A critical feature of its replication cycle is the reverse transcription step mediated by an error-prone viral polymerase that lacks proofreading activity (Bouckaert, Alvarado-Mora & Pinho, 2013; Delius et al., 1983; Girones & Miller, 1989; Orito et al., 1989). This results in a high mutation rate, facilitating the generation of extensive genetic diversity and enabling the virus to exist within a host as a complex mixture of closely related variants, known as a quasispecies.

This genetic heterogeneity has led to the classification of HBV into at least ten genotypes (A–J), defined by a divergence of >7.5% across the entire genome sequence (Pourkarim et al., 2014). These genotypes are further subdivided into subgenotypes based on a nucleotide divergence of 4–7.5% (Norder, Courouce & Magnius, 1994; Okamoto et al., 1988; Stuyver et al., 2000).

The geographical distribution of HBV genotypes is well-documented, with distinct patterns across different regions (Ahn, Yuen & Revill, 2009; Davies et al., 2013; Kimbi, Kramvis & Kew, 2004; Kramvis & Kew, 2007; Orito et al., 2001; Song, Cui & Kim, 2005). In China, a clear North-South gradient exists: genotype C is predominant in the north, while genotype B is more common in the south, though significant overlap and co-circulation occur in many areas (Xiao et al., 2023; Zeng et al., 2005). Thus, co-infection with both genotypes B and C may occasionally occur in a single host and might increase the incidence of genetic recombination.

This co-circulation creates opportunities for co-infection, where a single individual is infected with more than one HBV genotype. The clinical significance of HBV co-infection is increasingly recognized. Studies report a wide range of co-infection prevalence (7.9% to 67%), influenced by the sensitivity of detection methods (Andernach et al., 2012; Dopico et al., 2024; Wang et al., 2015). Critically, co-infection has been associated with higher viral loads, potentially reduced efficacy of antiviral therapies, and a more aggressive disease course, including a higher risk of progression to cirrhosis and HCC compared to mono-infections (Yin et al., 2008). However, analysis of individual strains from co-infected genotypes has been limited. Thus, the aim of this study was to clarify the genetic differences and the replication capacities of individual isolates of co-infected HBV strains.

However, a major gap in our understanding lies in the molecular characteristics and the individual replication dynamics of the distinct viral strains within a co-infected host. Are the observed severe clinical outcomes driven by one particularly aggressive genotype, a synergistic effect between genotypes, or the emergence of recombinant viruses? Disentangling the contribution of individual genotypes from a co-infection is technically challenging but essential for a precise understanding of viral pathogenesis.

A key viral genomic element influencing replication is the basal core promoter (BCP) of HBV. The double mutation Adenine (A)1762Thymine (T/)Guanine (G)1764A within the BCP region is a common mutation, frequently associated with enhanced viral replication and the development of advanced liver disease (Cui, Cho & Song, 2015; Lin et al., 2005). This mutation is more frequently observed in genotype C infections, which may partly explain its association with more severe clinical outcomes. However, the specific impact of this mutation can be context-dependent, varying with the viral genetic background (*e.g.*, genotype or subgenotype).

Therefore, the primary aim of this study was to isolate and characterize individual HBV strains from a patient co-infected with genotypes B and C. We sought to genetically characterize the isolates and, more importantly, to directly compare their intrinsic replication capacities in an isogenic cellular system. By using clones derived from a single patient, we controlled for host-specific factors, allowing for a direct comparison of viral fitness. Furthermore, we investigated the specific role of the BCP A1762T/G1764A double mutation in the replication phenotype of the isolated genotype C strain. This approach provides a unique opportunity to clarify the viral factors that may dictate disease progression in co-infected CHB patients, which could ultimately inform more personalized treatment strategies.

## Materials and Methods

### Serum HBV DNA isolation and determination of the HBV genotype

The HBV genome was isolated from 200 µL of serum collected from a 65-year-old male with CHB infection at the Second Affiliated Hospital of Hainan Medical University using a commercial viral DNA isolation kit (Qiagen, Hilden, Germany). The sample handling procedure was approved by the Ethics Committee of Hainan Medical University and the patient provided written informed consent to participate in this study. The HBV genotype was detected by nested polymerase chain reaction (PCR) using universal primers and genotype-specific primers designed by Naito, Hayashi & Abe (2001) (Table S1). In brief, HBV DNA isolated from serum was subjected to first-round PCR to amplify the regions spanning nucleotide (nt) positions 1–704 and 2,823–3,215 using universal primers. Second-round PCR was conducted using two µL of first-round PCR products as templates and a mixture of genotype-specific primers to determine the HBV genotype. The second-round PCR products were resolved by electrophoresis with 3% agarose gels. To avoid false-positive results, a negative control (without the DNA template) was included with each PCR assay.

### Amplification of the full-length HBV genome and Sanger sequencing

The full-length HBV genome was obtained by PCR with the primers F-1818 (*Sac* I) and L-reverse (*Sap* I) (Table S1). DNA polymerase (Toyobo, Osaka, Japan) with the proof-reading function was employed to minimize nucleotide mismatch during PCR amplification. The amplified full-length HBV genome was recovered from a 1% agarose gel and then ligated with a commercial cloning vector (Tiangen Biotech (Beijing) Co., Ltd., Beijing, China). Following transformation in *Escherichia coli* cells, colonies were subjected to conventional colony PCR using genotype-specific primers to verify successful cloning and identify the genotypes.

Colonies identified as genotypes B and C by PCR were cultured in Luria–Bertani (LB) medium supplemented with ampicillin (100 µg/mL). Plasmids (denoted as pLB-GtB and pLB-GtC, respectively) were isolated and subjected to DNA sequencing to confirm the genotype and subgenotype. The full-length HBV genome sequences were aligned with the ClustalW algorithm (https://www.theclustal.com/clustal2) along with reference sequences from the GenBank database (https://www.ncbi.nlm.nih.gov/genbank/) (Sayers et al., 2022; Thompson, Higgins & Gibson, 1994). A neighbor-joining phylogenetic tree was constructed to determine the subgenotype of HBV using Molecular Evolutionary Genetics Analysis (MEGA) software (version X; https://www.megasoftware.net/) (Kumar et al., 2018).

### HBV-expressing constructs and site-directed mutagenesis

A 189-bp fragment of HBV DNA spanning from nt 1799 to 1988 was amplified using the primer pairs S-forward (*Sap* I) and S-reverse (*Xho* I/*Hind* III) (Table S1), and was then ligated with the pLB vector. The pLB-GtB-c17 and pLB-GtC-c21 vectors were digested with the restriction enzymes *Sac* I and *Sap* I to recover the full-length HBV DNA, while plasmid DNA containing a 189-bp fragment was digested with the restriction enzymes *Hind* III and *Sap* I to obtain a short fragment of the HBV genome. Then, the recovered HBV DNA fragments were co-ligated with pCDNA 3.1(+), which was linearized by enzyme digestion with *Sac* I and *Hind* III, and verified by sequencing analysis. The constructed plasmid DNA was named pcDNA3.1-HBV GtB-c17 or pcDNA3.1-HBV GtC-c21, respectively.

PCR-based site-directed mutagenesis was performed using the QuikChange Site-Directed Mutagenesis Kit (Agilent Technologies, Inc., Santa Clara, CA, USA). For the genotype C clone, the T1762/A1764 nucleotides in pcDNA3.1-HBV GtC-c21 were reverted to A1762/G1764 to generate pcDNA3.1-HBV GtC-c21(WT). To further evaluate the effect of the BCP A1762T/G1764A double mutation in the genotype B background, the same mutation was introduced into pcDNA3.1-HBV GtB-c17 to generate pcDNA3.1-HBV GtB-c17(BCP). All mutant constructs were confirmed by Sanger sequencing before transfection.The primer pairs employed for mutagenesis are listed in Table S1.

### Cell cultivation and plasmid DNA transfection

Human hepatoblastoma (HepG2) cells were cultured in high-glucose Dulbecco’s modified Eagle’s medium supplemented with 10% fetal bovine serum (v/v) and penicillin (100 U/mL) and streptomycin (100 µg/mL) at 37 °C under an atmosphere of 5% CO_2_/95% air. HepG2 cells were seeded in 6-cm culture dishes and transiently transfected with four µg of plasmid DNA using Lipofectamine 3,000 transfection reagent (Thermo Fisher Scientific, Waltham, MA, USA). On post-transfection day 4, cells were harvested for analysis of HBV replication. All transient transfection experiments were independently conducted three times.

### Detection of viral replicative DNA intermediates and virion DNA

HBV replicative DNA intermediates within the cytoplasmic nucleocapsid (NC) were released by treatment with protease and sodium dodecyl sulfate (SDS), and identified by Southern blot analysis as previously described (Cui et al., 2013) and was further quantified by quantitative real-time PCR (qPCR). In brief, cells were lysed with NP-40 lysis buffer and centrifuged at 14,000 rpm for 5 min to isolate the NC from cytosol. Then, 20 µL of cytosolic lysate were incubated in a reaction mixture containing 1× DNase I buffer and 2 U of DNase I (New England Biolabs, Ipswich, MA, USA) at 37 °C for 1 h to remove DNA/RNA from outside the NC. The nuclease was inactivated by adding ethylene diamine tetraacetic acid (15 mM), and further incubated with SDS (0.5%) and proteinase K (0.5 µg) at 37 °C for 2 h. Then, the digested mixture was resolved by 1% agarose gel electrophoresis and transferred onto a nylon membrane. HBV replicative DNA intermediates on the membrane were probed with digoxigenin (DIG)-labeled HBV DNA probes (DIG High Prime DNA labeling and Detection Starter Kit II; Roche Diagnostics International Ltd., Rotkreuz, Switzerland). To avoid genotype-binding of the DNA probe, a 1:1 ratio of DNA probes targeting HBV genotypes B and C was used to detect both genotypes on the same membrane.

Secreted virion DNA were isolated from 200 µL of culture medium using a commercial viral DNA isolation kit (Qiagen, Hilden, Germany) and detected by quantitative real-time PCR described below.

### Quantitative real-time PCR

To quantify viral DNA copy number, a standard curve was generated using serial dilutions (1 ×10^4^–1 ×10^8^ copies/µL) of pcDNA3.1-HBV GtC-c21. HBV replicative intermediates and secreted viral DNA were detected a 25 µL of reaction consisting of 2x TB Green *Premix Ex Taq* II buffer (TB Green^®^ *Premix Ex Taq*™ II, Takara Bio Inc., Shiga, Japan), each primer at a final concentration of 0.4 µM, and 2 µL of DNA template. The thermal cycling protocol consisted of an initial denaturation at 95 °C for 3 min, followed by 40 cycles of denaturation at 95 °C for 15 s and annealing/extension at 60 °C for 30 s. Reaction specificity was confirmed by melting curve analysis. The absolute DNA copy number of each sample was calculated from the standard curve according to the corresponding Ct value. To minimize inter-experimental variation caused by differences in transfection efficiency, cell seeding density, or culture conditions, qPCR data were further normalized within each independent transfection experiment. In each experimental batch, the mean DNA copy number of the pcDNA3.1-HBV GtC-c21 or -c17 group was used as an internal reference and set to 1.0. The DNA copy numbers of all other samples from the same batch were divided by this reference value to generate relative fold-change values. These normalized values were then used for comparison across independent experiments. Primer sequences are listed in Table S1.

### Sequencing analysis

The full-length HBV DNA sequence alignment was conducted using the Clustal W method, followed by further determination of HBV genotypes/subgenotypes through maximum likelihood analysis with MEGA software, version X. The HBV full-genome sequences obtained in this study were compared with reference sequences from GenBank.

### Statistics analysis

All transient transfection experiments were independently repeated at least three times. For qPCR analysis, absolute HBV DNA copy numbers were first calculated from the standard curve and then normalized within each independent experiment as described above. The normalized relative values from three independent biological replicates were used for statistical analysis. Significant differences between two groups were evaluated using the independent Student’s *t*-test, and a probability (*p*) value <0.05 was considered statistically significant.

## Results

### Detection of co-infection by HBV genotypes B and C

By HBV genotype-specific nested PCR, two DNA bands migrated to the positions of genotype B (281 bp) and genotype C (122 bp), respectively (Fig. 1A, lane 2), implying either co-infection of both genotypes or genetic recombination of two genotypes into a single HBV genome, which might contain primer binding sites of both genotypes. For clarification, the HBV genome was amplified and subcloned into the pLB-vector. After bacterial transformation, 23 colonies were subjected to conventional colony PCR using genotype-specific primers (Fig. 1B). The results of colony PCR identified 10 (43.4%, 10/23) colonies with a single DNA band, which included 6 (60%, 6/10) colonies that were genotype B and 4 (40%, 4/10) that were genotype C (Fig. 1B).

**Figure 1:**
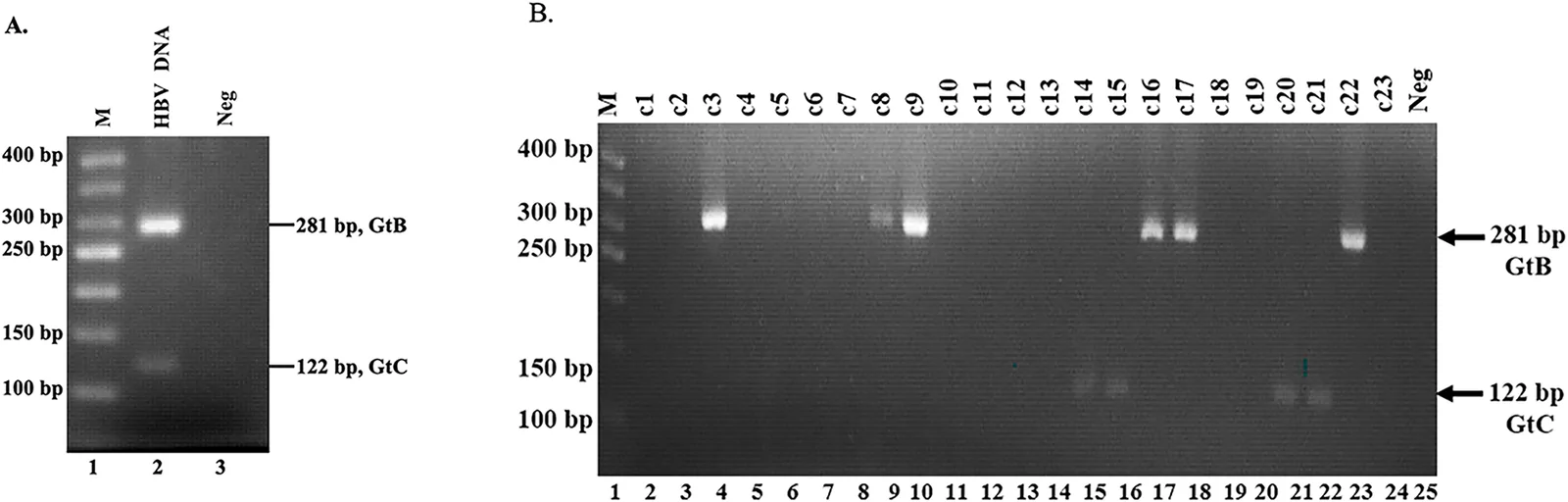
Determination of the HBV genotype by genotype-specific PCR. (A) HBV DNA isolated from 200 µL of serum was amplified by nested PCR with genotype-specific primers. The PCR products were resolved on 3% agarose gels. (B) A total of 23 colonies were subjected to conventional colony PCR with second-round genotype-specific primers to identify positive clones. The PCR products were resolved on 3% agarose gels. Abbreviations: GtB, genotype B; GtC, genotype C; M, DNA marker; Neg, negative control.

All PCR-positive clones were subjected to Sanger sequencing analysis. The obtained full-length HBV sequences were analyzed by phylogenetic tree construction to determine HBV subgenotype. Phylogenetic analysis revealed that all clones were classified into subgenotype C1, or subgenotype B2, respectively (Fig. 2). The nucleotide identity of full-length HBV DNA sequences was 100% and 99.9% within B2 and C1 groups, respectively. None of the clones harbored nucleotide mutations known to impair viral replication or confer drug resistance. Therefore, one representative clone from each genotype group (c17 from genotype B2 and c21 from genotype C1) was randomly selected for subsequent analyses. The c17 and c21 sequences were deposited into the GenBank database under the accession numbers MW497299 and MW755445, respectively. The detailed information of full-length HBV DNA sequence of all clones was listed in supplementary data.

**Figure 2:**
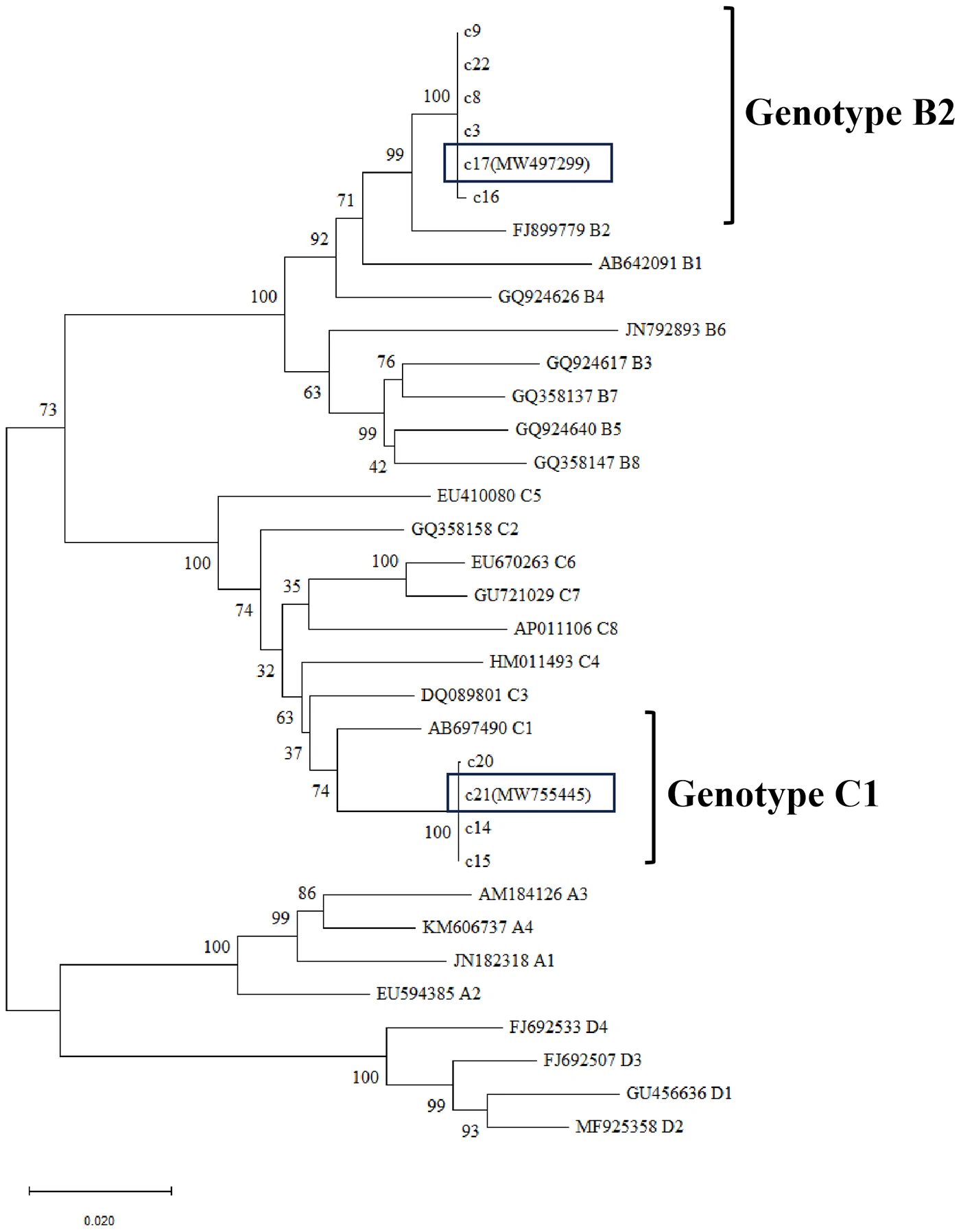
Verification of genotype/subgenotype by phylogenetic analysis. Full-length HBV DNA was amplified and subcloned into the pLB-Vector. After transformation, plasmid DNA was isolated and subjected to sequencing analysis. A phylogenetic tree was constructed based on the maximum-likelihood method. The studied sequence is highlighted with a rectangular box.

### Comparative analysis of HBV replication capacity between genotype B genotype C

To compare the viral replication capacity between genotype B and C, HepG2 cells were transiently transfected with the HBV-expressing plasmids pcDNA3.1-HBV GtB-c17 and pcDNA3.1-HBV GtC-c21, which were constructed using the full-length sequences of c17 and c21, respectively. The cytoplasmic viral replicative intermediates were detected by Southern blot and quantified by qPCR. Virion-associated DNA was isolated from cell culture supernatants and quantified by qPCR to assess virion secretion levels. As shown in Fig. 3, pcDNA3.1-HBV GtC-c21 demonstrated significantly higher levels of HBV replicative intermediates (Figs. 3A and 3B) and virion secretion (Fig. 3C) compared to pcDNA3.1-HBV GtB-c17, indicating superior replication capacity of genotype C clone c21 relative to genotype B clone c17.

**Figure 3:**
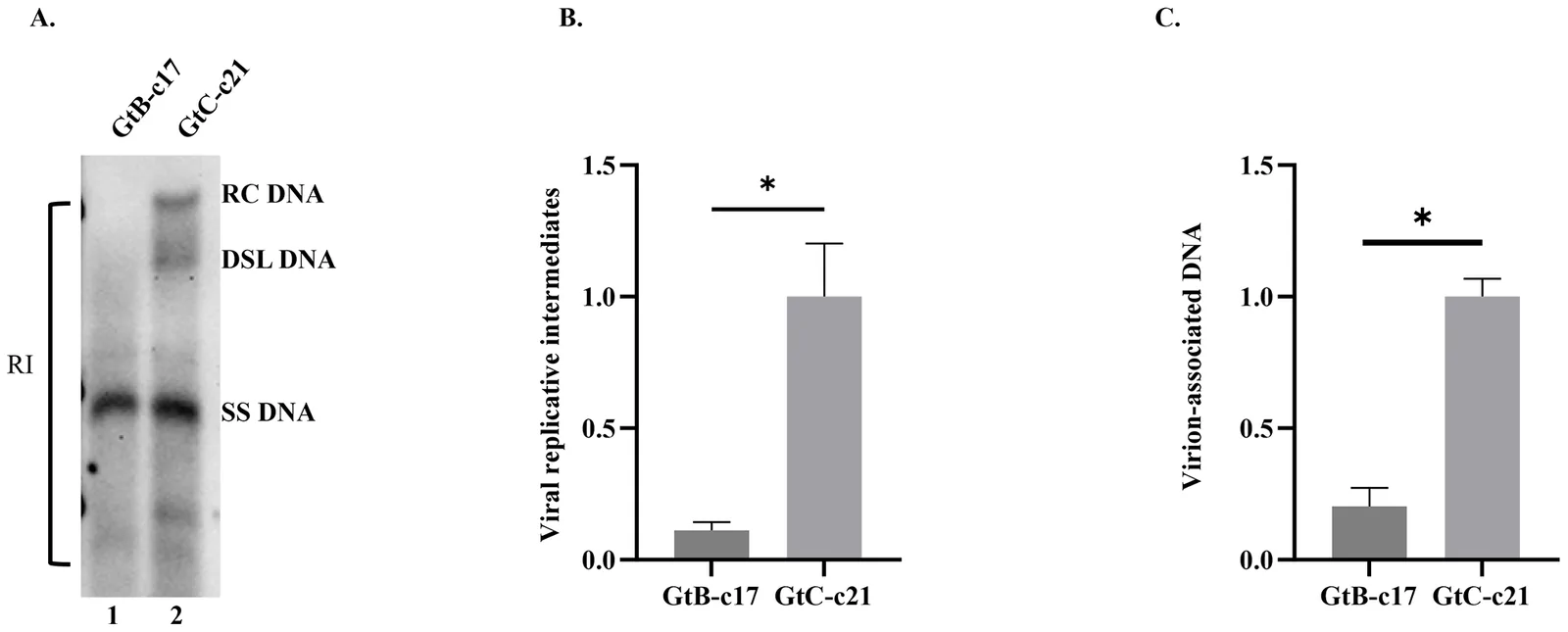
Comparison of viral replication capacity between genotypes B2 and C1. (A) HepG2 cells were transiently transfected with plasmid DNA harboring genes of genotypes B2 and C1, and HBV replicative intermediates were detected by Southern blot analysis. (B) Viral replicative intermediates and (C) Virion-associated DNA were quantified by qPCR and the significance of differences of the level of viral replication between the two groups was analyzed with the independent Student’s *t*-test (* *p* < 0.05). Abbreviations: DSL, double stranded linear; RC, relaxed circular; RI, replicative intermediates; SS, single stranded.

### BCP mutation did not enhance the viral replication

Sequence analysis revealed the presence of two common BCP mutations (A1762T and G1764A) in c21. These mutations have been previously reported to enhance viral replication (Cui, Cho & Song, 2015; Lindh et al., 1999; Yang et al., 2023). To investigate whether the double mutation contributed the observed higher replication capacity of c21 than c17, pcDNA3.1-HBV GtC-c21(WT) was generated by site-directed mutagenesis to revert the BCP mutation to the wild-type sequence. Intriguingly, transient transfection of HepG2 cells with pcDNA3.1-HBV GtC-c21(WT) resulted slightly higher levels of viral replicative intermediates (Figs. 4A and 4B) and virion secretion (Fig. 4C) compared to pcDNA3.1-HBV GtC-c21 harboring the BCP double mutation, suggesting that the BCP mutations did not account for the enhanced replication capacity of genotype C clone.

**Figure 4:**
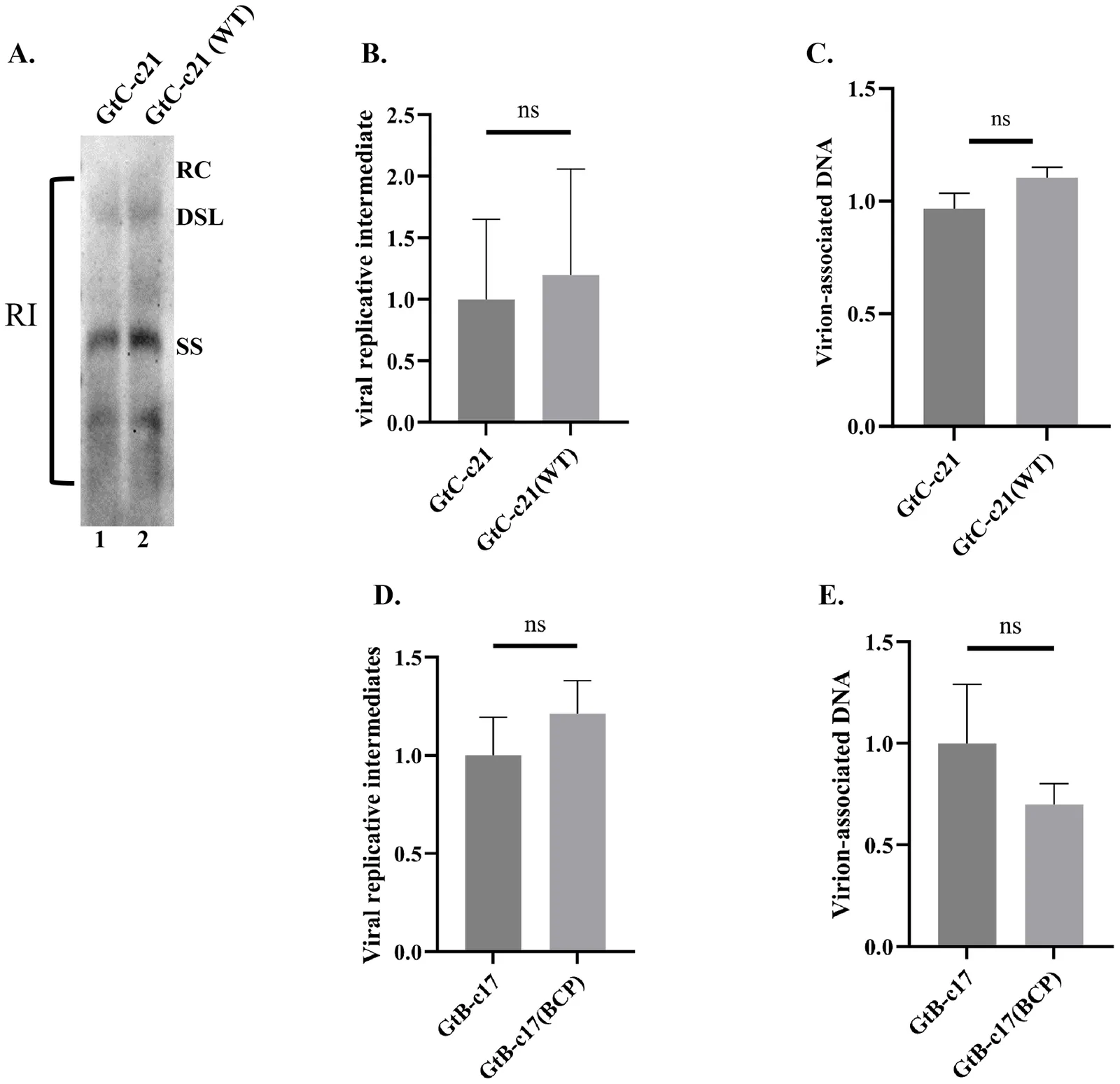
Effect of BCP A1762T/G1764A mutation on viral replication. (A) HepG2 cells were transiently transfected with the genotype C clone GtC-c21 or its BCP reversion mutant, and intracellular viral replicative intermediates were detected by Southern blot analysis. (B) Intracellular viral replicative intermediates and (C) virion-associated HBV DNA were quantified by qPCR. (D) Intracellular viral replicative intermediates and (E) virion-associated HBV DNA from cells transfected with GtB-c17 or GtB-c17(BCP) were quantified by qPCR. Statistical significance was analyzed using the independent Student’s *t*-test, and *p* < 0.05 was considered statistically significant. No statistically significant difference was observed between GtB-c17 and GtB-c17(BCP). Abbreviations: BCP, basal core promoter; DSL, double-stranded linear; RC, relaxed circular; RI, replicative intermediates; SS, single-stranded.

To further determine whether the effect of A1762T/G1764A was dependent on the viral genetic background, we introduced the same BCP double mutation into the genotype B2 clone c17 and generated pcDNA3.1-HBV GtB-c17(BCP). HepG2 cells were transiently transfected with pcDNA3.1-HBV GtB-c17 or pcDNA3.1-HBV GtB-c17(BCP), and intracellular viral replicative intermediates, and virion-associated DNA were examined. Consistently, qPCR analysis showed that intracellular viral replicative intermediate DNA was slightly higher in the GtB-c17(BCP) group than in the parental GtB-c17 group. However, the differences in intracellular viral replicative intermediate DNA did not reach statistical significance. In contrast, virion-associated DNA in the culture supernatant showed a slight decrease in the GtB-c17(BCP) group compared with the parental GtB-c17 group, but this difference was also not statistically significant. Together, these results indicate that introduction of the A1762T/G1764A mutation into the genotype B2 clone did not significantly enhance HBV replication or virion secretion under our experimental conditions.

## Discussion

This study successfully isolated and characterized two distinct HBV strains, subgenotypes B2 and C1, from a single CHB patient, confirming a true co-infection. The key finding is that the genotype C1 isolate exhibited a significantly greater replication capacity than the genotype B2 isolate in a controlled cell culture system. This intrinsic replication advantage of genotype C may contribute to the more severe clinical outcomes often associated with this genotype in mono-infection settings and could be a critical driver of pathogenesis in co-infected individuals.

The high replication rate of HBV, producing up to 10^11^ virions daily, coupled with its error-prone replication mechanism, fosters significant genetic diversity and enables the coexistence of multiple genotypes in a single geographical region (Norder et al., 1993; Nowak et al., 1996). This environment predisposes individuals to co-infections, which are not merely a virological curiosity but have significant clinical implications, including potential treatment failure and accelerated disease progression (Hannoun et al., 2002). Our detection of both B2 and C1 subgenotypes in a patient from Hainan, a region with documented genotype overlap, aligns with this epidemiological context (Xiao et al., 2023). Such co-infections are a prerequisite for genetic recombination, a known evolutionary mechanism in HBV that can give rise to viable recombinant viruses with potentially altered phenotypes (Locarnini et al., 2013; Sugauchi et al., 2002). While our colony PCR and phylogenetic analysis confirmed the presence of two distinct genotypes without evidence of recombination in the studied clones, the possibility of recombinant species existing within the patient’s quasispecies cannot be ruled out.

The most striking result of our functional analysis was the superior replication capacity of the genotype C1 clone compared to the genotype B2 clone. This finding provides a plausible virological explanation for the observed aggressive disease course in many patients infected with or co-infected with genotype C. It suggests that in a co-infection scenario, genotype C may outcompete genotype B over time, potentially leading to genotype dominance that could influence the patient’s response to therapy and long-term prognosis.

Intriguingly, our investigation into the BCP A1762T/G1764A double mutation, which was present in the genotype C1 clone, yielded a counterintuitive result. Contrary to numerous reports that this mutation enhances viral replication, reverting the mutation to the wild-type sequence in our genotype C1 clone unexpectedly resulted in a further increase in replication capacity (Cui, Cho & Song, 2015; Yang et al., 2023). This indicates that the A1762T/G1764A mutation was not the primary driver of the high replication phenotype phenotype of this genotype C1 clone.

To further evaluate whether the effect of the BCP double mutation was influenced by the viral genetic background, we introduced A1762T/G1764A into the genotype B2 clone c17. In this genotype B background, the BCP-mutated clone showed a non-significant trend toward increased intracellular viral replicative intermediate DNA, whereas virion-associated DNA showed a non-significant decreasing trend. These data suggest that the BCP double mutation alone is insufficient to significantly enhance the overall replication and secretion phenotype of the genotype B2 clone c17. The different trends observed for intracellular replication markers and extracellular virion-associated DNA also imply that the BCP mutation may affect distinct steps of the viral life cycle in a context-dependent manner, including transcription, nucleocapsid-associated DNA synthesis, or virion release.

These findings support the concept that the biological effect of A1762T/G1764A is highly dependent on the complete viral genomic context rather than being determined solely by the two nucleotide substitutions. Previous studies, including transcriptomic-level analyses, have shown that A1762T/G1764A can alter HBV RNA profiles and thereby influence replication (Yang et al., 2023). However, in naturally occurring full-length HBV isolates, additional sequence variations in regulatory regions or coding sequences, including Core, Polymerase, and envelope proteins, may modulate or even override the effect of the BCP mutation. Therefore, the superior replication capacity of the genotype C1 clone observed in this study is more likely attributable to other genotype- or isolate-specific genomic features, or to the combined effects of multiple viral regions, rather than to the A1762T/G1764A mutation alone.

Several limitations of this study should be acknowledged. First, this study was based on viral clones isolated from a single patient co-infected with HBV genotypes B and C. Although this design allowed a direct comparison of genotype-specific replication capacities under the same host background, the findings may not fully represent the broader genetic diversity of HBV co-infections. Therefore, validation using a larger cohort of patients and additional genotype B and C isolates is required to determine the generalizability of the observed replication advantage of the genotype C1 clone. Second, our functional analysis focused mainly on viral DNA replication and virion-associated DNA production in HepG2 cells. We did not perform transcriptomic-level analyses, such as quantification of pgRNA, precore RNA, or subgenomic transcripts, which could provide further mechanistic insight into the regulation of viral replication. Third, although we specifically examined the contribution of the well-characterized BCP A1762T/G1764A mutation, other sequence variations in the genotype C1 clone, including mutations affecting viral regulatory regions or proteins such as Core, Polymerase, and envelope proteins, may also contribute to its replication phenotype. Systematic mutational mapping and protein-level analyses will be needed to clarify these potential contributions. Finally, HepG2 cells provide a convenient and reproducible *in vitro* system for comparing HBV replication, but they do not fully recapitulate the physiological hepatic microenvironment or host immune responses *in vivo*. Future studies using more physiologically relevant models, together with larger numbers of patient-derived isolates, will be important to further define the virological and pathogenic significance of genotype-specific replication differences in HBV co-infection.

In conclusion, by isolating and characterizing individual HBV strains from a co-infected patient, we have directly demonstrated that genotype C1 possesses a greater inherent replication capacity than genotype B2, independent of the BCP A1762T/G1764A mutation. This study underscores the importance of analyzing the properties of individual viral components within a co-infection. Understanding such genotype-specific behaviors is crucial for advancing our knowledge of HBV pathogenesis and moving toward more precise virological monitoring and personalized treatment strategies for CHB patients.

## Supplemental Information

10.7717/peerj.21866/supp-1 Supplemental Information 1 List of PCR primer pairs

10.7717/peerj.21866/supp-2 Supplemental Information 2 HBV full-length genome sequence

10.7717/peerj.21866/supp-3 Supplemental Information 3 Uncropped blot pictures

10.7717/peerj.21866/supp-4 Supplemental Information 4 Raw data (qPCR)

10.7717/peerj.21866/supp-5 Supplemental Information 5 MIQE checklist

## Additional Information and Declarations

### Data Availability

The following information was supplied regarding data availability: The raw data is available in the Supplemental Files. The data are available at GenBank: MW497299.1, MW755445.1.
