# Supplementary material for: Intrinsic replication advantage of a Hepatitis B Virus genotype C strain is independent of the basal core promoter A1762T/G1764A mutation
Source: PeerJ. 2026 Nov 5;14:e21866. doi: 10.7717/peerj.21866 (PMC13744702; doi:10.7717/peerj.21866)
Supplement: Supplementary file 1 [file peerj-14-21866-s001.docx]

**Table 1. List of PCR primer pairs**

|  | **Primers** | **Sequence (5’→3’)** | **Nucleotide position** |
| --- | --- | --- | --- |
| **Genotypic PCR** |  |  |  |
| 1^st^ round | P1 | 5'-TCACCATATTCTTGGGAACAAGA-3' | nt 2823-2845 |
|  | S1-2 | 5'-CGAACCACTGAACAAATGGC-3' | nt 685-704 |
| 2^nd^ round | Reverse genotype A | 5'-CTCGCGGAGATTGACGAGATGT-3' | nt 113-134 |
|  | Reverse genotype B | 5'-CAGGTTGGTGAGTGACTGGAGA-3' | nt 324-345 |
|  | Reverse genotype C | 5'-GGTCCTAGGAATCCTGATGTTG-3' | nt 165-186 |
|  | Forward genotype ABC | 5'-GGCTCMAGTTCMGGAACAAGT-3' | nt 67-86 |
| **HBV Plasmid** |  |  |  |
| Full length | F-1818(*Sac*I) | 5'-CTATATTAAGCAGAGCTCCACCAGCACCATGCAACTTTTTC-3' | nt 1804-1826 |
|  | L-Reverse(*Sap*I) | 5'-CCGGAAAGCTTGAGCTCTTCAACACACCAATTTATGCCTAC-3 | nt 1783-1803 |
| Short fragment | S-Forward(*Sap*I) | 5'-CCGGAAAGCTTGAGCTCTTCGTGTTCACCAGCACCATGCA-3 | nt 1799-1818 |
|  | S-Reverse(*Xho*I/*Hin*dIII) | 5'-GATTCCCTCGAGAAGCTTGATCTCGAACAGAAGGAAAGAA-3' | nt 1967-1988 |
| **For sequencing** |  |  |  |
|  | CMV-F | 5'-CGCAAATGGGCGGTAGGCGTG-3' | pcDNA 3.1 |
|  | P4 | 5'-CCTTGGACACATAAGGTGGG-3' | nt 2457-2476 |
|  | P5 | 5'-GTGGAGCCCTCAGGCTCAGG-3' | nt 3075-3094 |
|  | P6 | 5'-GCTGCTATGCCTCATCTTC-3' | nt 415-433 |
|  | P7 | 5'-CCAACTTACAAGGCCTTTC-3' | nt 1102-1120 |
|  | BGH | 5'-CAGGGTCAAGGAAGGCAC-3' | pCDNA 3.1 |
| **For mutagenesis** |  |  |  |
|  | 1762/1764 Forward | 5’-GGGGAGGAGATTAGGTTAAAGGTCTTTGTACTAGGAGG-3' | nt 1743-1780 |
|  | 1762/1764 Reverse | 5’-CCTCCTAGTACAAAGACCTTTAACCTAATCTCCTCCCC-3’ | nt 1743-1780 |
|  | 1762/1764 BCP Forward | 5’-TAGGTTAATGATCTTTGTACTAGGAGGCT-3' | nt 1743-1780 |
|  | 1762/1764 BCP Reverse | 5’-TACAAAGATCATTAACCTAATCTCCTCC-3’ | nt 1743-1780 |
|  |  |  |  |
| **For qPCR** |  |  |  |
|  | Q1 forward | 5'-TCTAGACTCGTGGTGGACTTCTCTC-3’ | nt 249~273 |
|  | Q2 Reverse | 5'-CATAGCAGCAGGATGMARAGGAA-3' | nt 403~425 |
