# Supplementary material for: Intrinsic replication advantage of a Hepatitis B Virus genotype C strain is independent of the basal core promoter A1762T/G1764A mutation
Source: PeerJ. 2026 Nov 5;14:e21866. doi: 10.7717/peerj.21866 (PMC13744702; doi:10.7717/peerj.21866)
Supplement: Supplementary file 2 [file peerj-14-21866-s002.docx]

**Genotype B**

**c3:**

CTCCACCACTTTCCACCAAACTCTTCAAGATCCAAGAGTCAGGGCCCTGTACTTTCCTGCTGGTGGCTCCAGTTCAGGAACAGTGAGCCCTGCTCAGAATACTGTCTCTGCCATATCGTCAATCTTATCGAAGACTGGGGACCCTGTACCGAACATGGAGAACATCGCATCAGGACTCCTAGGACCCCTGCTCGTGTTACAGGCGGGGTTTTTCTTGTTGACAAAAATCCTCACAATACCACAGAGTCTAGACTCGTGGTGGACTTCTCTCAATTTTCTAGGGGGAACACCCGTGTGTCTTGGCCAAAATTCGCAGTCCCAAATCTCCAGTCACTCACCAACCTGTTGTCCTCCAATTTGTCCTGGTTATCGCTGGATGTGTCTGCGGCGTTTTATCATCTTCCTTTGCATCCTGCTGCTATGCCTCATCTTCTTGTTGGTTCTTCTGGACTATCAAGGTATGTTGCCCGTTTGTCCTCTAATTCCAGGATCATCAACAACCAGCACCGGACCATGCAAAACCTGCACGACTCCTGCTCAAGGAACCTCTATGTTTCCCTCATGTTGCTGTACAAAACCTACGGACGGAAACTGCACCTGTATTCCCATCCCATCATCTTGGGCTTTCGCAAAATACCTATGGGAGTGGGCCTCAGTCCGTTTCTCTTGGCTCAGTTTACTAGTGCCATTTGTTCAGTGGTTCGTAGGGCTTTCCCCCACTGTCTGGCTTTCAGTTATATGGATGATGTGGTATTGGGGGCCAAGTCTGTACAACATCTTGAGTCCCTTTATGCCGCTGTTACCAATTTTCTTTTGTCTTTGGGTATACATTTAAACCCTCACAAAACAAAAAGATGGGGATATTCCCTTAACTTCATGGGCTATGTAATTGGGTGTTGGGGCACATTGCCACAGGAACATATTGTACAAAAAATCAAAATGTGTTTTAGAAAACTTCCTGTAAACAGACCTATTGATTGGAAAGTATGTCAACGAATTGTGGGTCTTTTGGGGTTTGCCGCCCCTTTCACGCAATGTGGATATCCTGCTTTAATGCCTTTATATGCATGTATACAAGCAAAACAGGCTTTTACTTTCTCGCCAACTTACAAGGCCTTTCTGAGTAAACAGTATCTGAACCTTTACCCCGTTGCTCGGCAACGGTCTGGTCTGTGCCAAGTGTTTGCTGACGCAACCCCCACTGGTTGGGGCTTGGCCATAGGCCATCAGCGCATGCGTGGAACCTTTGTGTCTCCTCTGCCGATCCATACTGCGGAACTCCTAGCCGCTTGTTTTGCTCGCAGCAGGTCTGGGGCAAAACTCATCGGGACTGACAATTCTGTCGTGCTCTCCCGCAAGTATACATCGTTCCCATGGCTGCTAGGCTGTGCTGCCAACTGGATCCTGCGCGGGACGTCCTTTGTTTACGTCCCGTCGGCGCTGAATCCCGCGGACGACCCCTCCCGGGGCCGCTTGGGGCTCTACCGCCCGCTTCTCCGCCTGTTCTACCGACCGACCACGGGGCGTACCTCTCTTTACGCGGACTCCCCGTCTGTGCCTTCTCATCTGCCGGACCGTGTGCACTTCGCTTCACCTCTGCACGTCGCATGGAGACCACCGTGAACGCCCACCGGAACTTGCCCAAGGTCTTGCATAAGAGGACTCTTGGACTTTCAGCAATGTCAACGACCGACCTTGAGGCATACTTCAAAGACTGTGTGTTTACTGAGTGGGAGGAGTTGGGGGAGGAGATTAGGTTAAAGGTCTTTGTACTAGGAGGCTGTAGGCATAAATTGGTGTGTTCACCAGCACCATGCAACTTTTTCACCTCTGCCTAATCATCTCATGTTCATGTCCTACTGTTCAAGCCTCCAAGCTGTGCCTTGGGTGGCTTTGGGGCATGGACATTGACACCTATAAAGAATTTGGAGCTTCTGTGGAGTTACTCTCTTTTTTGCCTTCTGACTTCTTTCCTTCTGTTCGAGATCTCCTCGACACCGCCTCTGCTCTGTATCGGGAGGCCTTAGAGTCTCCGGAACATTGTTCACCTCACCATACGGCACTCAGGCAAGCTATTGTGTGTTGGGGTGAGTTGATGAATCTAGCCACCTGGGTGGGAAGTAATTTGGAAGATCCAGCATCCAGGGAATTAGTAGTCAGCTATGTCAACGTTAATATGGGCCTAAAAATCAGACAACTATTGTGGTTTCACATTTCCTGTCTTACTTTTGGGAGAGAAACTGTTCTTGAATATTTGGTGTCTTTTGGAGTGTGGATTCGCACTCCTCCTGCATATAGACCACCAAATGCCCCTATCTTATCAACACTTCCGGAAACTACTGTTGTTAGACGAAGAGGCAGGTCCCCTAGAAGAAGAACTCCCTCGCCTCGCAGACGAAGGTCTCAATCGCCGCGTCGCAGAAGATCTCAATCTCGGGAATCTCAATGTTAGTATTCCTTGGACACATAAGGTGGGAAACTTTACGGGGCTTTATTCTTCTACGGTACCTTGCTTTAATCCTAAATGGCAAACTCCTTCTTTTCCTGACATTCATTTGCAGGAGGACATTGTTGATAGATGTAAGCAATTTGTGGGGCCCCTTACAGTAAATGAAAACAGGAGACTAAAATTAATTATGCCTGCTAGGTTTTATCCCAATGTTACTAAATATTTGCCCTTAGATAAAGGGATCAAACCTTATTATCCAGAGCATGTAGTTAATCATTACTTCCAGACGAGACATTATTTACACACTCTTTGGAAGGCGGGTATCTTATATAAAAGAGAGTCCACACGTAGCGCCTCATTTTGCGGGTCACCATATTCTTGGGAACAAGATCTACAGCATGGGAGGTTGGTCTTCCAAACCTCGAAAAGGCATGGGGACAAATCTTTCTGTCCCCAATCCCCTGGGATTCTTCCCCGATCATCAGTTGGACCCTGCATTCAAAGCCAACTCAGAAAATCCAGATTGGGACCTCAACCCGCACAAGGACAACTGGCCGGACGCCAACAAGGTGGGAGTGGGAGCATTCGGGCCAGGGTTCATCCCTCCCCATGGGGGACTGTTGGGGTGGAGCCCTCAGGCTCAGGGCATACTCACATCTGTGCCAGCAGCTCCTCCTCCTGCCTCCACCAATCGGCAGTCAGGAAGGCAGCCTACTCCCTTATCTCCACCTCTAAGGGACACTCATCCTCAGGCCATACAGTGGAA

**c8:**

CTCCACCACTTTCCACCAAACTCTTCAAGATCCAAGAGTCAGGGCCCTGTACTTTCCTGCTGGTGGCTCCAGTTCAGGAACAGTGAGCCCTGCTCAGAATACTGTCTCTGCCATATCGTCAATCTTATCGAAGACTGGGGACCCTGTACCGAACATGGAGAACATCGCATCAGGACTCCTAGGACCCCTGCTCGTGTTACAGGCGGGGTTTTTCTTGTTGACAAAAATCCTCACAATACCACAGAGTCTAGACTCGTGGTGGACTTCTCTCAATTTTCTAGGGGGAACACCCGTGTGTCTTGGCCAAAATTCGCAGTCCCAAATCTCCAGTCACTCACCAACCTGTTGTCCTCCAATTTGTCCTGGTTATCGCTGGATGTGTCTGCGGCGTTTTATCATCTTCCTTTGCATCCTGCTGCTATGCCTCATCTTCTTGTTGGTTCTTCTGGACTATCAAGGTATGTTGCCCGTTTGTCCTCTAATTCCAGGATCATCAACAACCAGCACCGGACCATGCAAAACCTGCACGACTCCTGCTCAAGGAACCTCTATGTTTCCCTCATGTTGCTGTACAAAACCTACGGACGGAAACTGCACCTGTATTCCCATCCCATCATCTTGGGCTTTCGCAAAATACCTATGGGAGTGGGCCTCAGTCCGTTTCTCTTGGCTCAGTTTACTAGTGCCATTTGTTCAGTGGTTCGTAGGGCTTTCCCCCACTGTCTGGCTTTCAGTTATATGGATGATGTGGTATTGGGGGCCAAGTCTGTACAACATCTTGAGTCCCTTTATGCCGCTGTTACCAATTTTCTTTTGTCTTTGGGTATACATTTAAACCCTCACAAAACAAAAAGATGGGGATATTCCCTTAACTTCATGGGCTATGTAATTGGGTGTTGGGGCACATTGCCACAGGAACATATTGTACAAAAAATCAAAATGTGTTTTAGAAAACTTCCTGTAAACAGACCTATTGATTGGAAAGTATGTCAACGAATTGTGGGTCTTTTGGGGTTTGCCGCCCCTTTCACGCAATGTGGATATCCTGCTTTAATGCCTTTATATGCATGTATACAAGCAAAACAGGCTTTTACTTTCTCGCCAACTTACAAGGCCTTTCTGAGTAAACAGTATCTGAACCTTTACCCCGTTGCTCGGCAACGGTCTGGTCTGTGCCAAGTGTTTGCTGACGCAACCCCCACTGGTTGGGGCTTGGCCATAGGCCATCAGCGCATGCGTGGAACCTTTGTGTCTCCTCTGCCGATCCATACTGCGGAACTCCTAGCCGCTTGTTTTGCTCGCAGCAGGTCTGGGGCAAAACTCATCGGGACTGACAATTCTGTCGTGCTCTCCCGCAAGTATACATCGTTCCCATGGCTGCTAGGCTGTGCTGCCAACTGGATCCTGCGCGGGACGTCCTTTGTTTACGTCCCGTCGGCGCTGAATCCCGCGGACGACCCCTCCCGGGGCCGCTTGGGGCTCTACCGCCCGCTTCTCCGCCTGTTCTACCGACCGACCACGGGGCGTACCTCTCTTTACGCGGACTCCCCGTCTGTGCCTTCTCATCTGCCGGACCGTGTGCACTTCGCTTCACCTCTGCACGTCGCATGGAGACCACCGTGAACGCCCACCGGAACTTGCCCAAGGTCTTGCATAAGAGGACTCTTGGACTTTCAGCAATGTCAACGACCGACCTTGAGGCATACTTCAAAGACTGTGTGTTTACTGAGTGGGAGGAGTTGGGGGAGGAGATTAGGTTAAAGGTCTTTGTACTAGGAGGCTGTAGGCATAAATTGGTGTGTTCACCAGCACCATGCAACTTTTTCACCTCTGCCTAATCATCTCATGTTCATGTCCTACTGTTCAAGCCTCCAAGCTGTGCCTTGGGTGGCTTTGGGGCATGGACATTGACACCTATAAAGAATTTGGAGCTTCTGTGGAGTTACTCTCTTTTTTGCCTTCTGACTTCTTTCCTTCTGTTCGAGATCTCCTCGACACCGCCTCTGCTCTGTATCGGGAGGCCTTAGAGTCTCCGGAACATTGTTCACCTCACCATACGGCACTCAGGCAAGCTATTGTGTGTTGGGGTGAGTTGATGAATCTAGCCACCTGGGTGGGAAGTAATTTGGAAGATCCAGCATCCAGGGAATTAGTAGTCAGCTATGTCAACGTTAATATGGGCCTAAAAATCAGACAACTATTGTGGTTTCACATTTCCTGTCTTACTTTTGGGAGAGAAACTGTTCTTGAATATTTGGTGTCTTTTGGAGTGTGGATTCGCACTCCTCCTGCATATAGACCACCAAATGCCCCTATCTTATCAACACTTCCGGAAACTACTGTTGTTAGACGAAGAGGCAGGTCCCCTAGAAGAAGAACTCCCTCGCCTCGCAGACGAAGGTCTCAATCGCCGCGTCGCAGAAGATCTCAATCTCGGGAATCTCAATGTTAGTATTCCTTGGACACATAAGGTGGGAAACTTTACGGGGCTTTATTCTTCTACGGTACCTTGCTTTAATCCTAAATGGCAAACTCCTTCTTTTCCTGACATTCATTTGCAGGAGGACATTGTTGATAGATGTAAGCAATTTGTGGGGCCCCTTACAGTAAATGAAAACAGGAGACTAAAATTAATTATGCCTGCTAGGTTTTATCCCAATGTTACTAAATATTTGCCCTTAGATAAAGGGATCAAACCTTATTATCCAGAGCATGTAGTTAATCATTACTTCCAGACGAGACATTATTTACACACTCTTTGGAAGGCGGGTATCTTATATAAAAGAGAGTCCACACGTAGCGCCTCATTTTGCGGGTCACCATATTCTTGGGAACAAGATCTACAGCATGGGAGGTTGGTCTTCCAAACCTCGAAAAGGCATGGGGACAAATCTTTCTGTCCCCAATCCCCTGGGATTCTTCCCCGATCATCAGTTGGACCCTGCATTCAAAGCCAACTCAGAAAATCCAGATTGGGACCTCAACCCGCACAAGGACAACTGGCCGGACGCCAACAAGGTGGGAGTGGGAGCATTCGGGCCAGGGTTCATCCCTCCCCATGGGGGACTGTTGGGGTGGAGCCCTCAGGCTCAGGGCATACTCACATCTGTGCCAGCAGCTCCTCCTCCTGCCTCCACCAATCGGCAGTCAGGAAGGCAGCCTACTCCCTTATCTCCACCTCTAAGGGACACTCATCCTCAGGCCATACAGTGGAA

**c9:**

CTCCACCACTTTCCACCAAACTCTTCAAGATCCAAGAGTCAGGGCCCTGTACTTTCCTGCTGGTGGCTCCAGTTCAGGAACAGTGAGCCCTGCTCAGAATACTGTCTCTGCCATATCGTCAATCTTATCGAAGACTGGGGACCCTGTACCGAACATGGAGAACATCGCATCAGGACTCCTAGGACCCCTGCTCGTGTTACAGGCGGGGTTTTTCTTGTTGACAAAAATCCTCACAATACCACAGAGTCTAGACTCGTGGTGGACTTCTCTCAATTTTCTAGGGGGAACACCCGTGTGTCTTGGCCAAAATTCGCAGTCCCAAATCTCCAGTCACTCACCAACCTGTTGTCCTCCAATTTGTCCTGGTTATCGCTGGATGTGTCTGCGGCGTTTTATCATCTTCCTTTGCATCCTGCTGCTATGCCTCATCTTCTTGTTGGTTCTTCTGGACTATCAAGGTATGTTGCCCGTTTGTCCTCTAATTCCAGGATCATCAACAACCAGCACCGGACCATGCAAAACCTGCACGACTCCTGCTCAAGGAACCTCTATGTTTCCCTCATGTTGCTGTACAAAACCTACGGACGGAAACTGCACCTGTATTCCCATCCCATCATCTTGGGCTTTCGCAAAATACCTATGGGAGTGGGCCTCAGTCCGTTTCTCTTGGCTCAGTTTACTAGTGCCATTTGTTCAGTGGTTCGTAGGGCTTTCCCCCACTGTCTGGCTTTCAGTTATATGGATGATGTGGTATTGGGGGCCAAGTCTGTACAACATCTTGAGTCCCTTTATGCCGCTGTTACCAATTTTCTTTTGTCTTTGGGTATACATTTAAACCCTCACAAAACAAAAAGATGGGGATATTCCCTTAACTTCATGGGCTATGTAATTGGGTGTTGGGGCACATTGCCACAGGAACATATTGTACAAAAAATCAAAATGTGTTTTAGAAAACTTCCTGTAAACAGACCTATTGATTGGAAAGTATGTCAACGAATTGTGGGTCTTTTGGGGTTTGCCGCCCCTTTCACGCAATGTGGATATCCTGCTTTAATGCCTTTATATGCATGTATACAAGCAAAACAGGCTTTTACTTTCTCGCCAACTTACAAGGCCTTTCTGAGTAAACAGTATCTGAACCTTTACCCCGTTGCTCGGCAACGGTCTGGTCTGTGCCAAGTGTTTGCTGACGCAACCCCCACTGGTTGGGGCTTGGCCATAGGCCATCAGCGCATGCGTGGAACCTTTGTGTCTCCTCTGCCGATCCATACTGCGGAACTCCTAGCCGCTTGTTTTGCTCGCAGCAGGTCTGGGGCAAAACTCATCGGGACTGACAATTCTGTCGTGCTCTCCCGCAAGTATACATCGTTCCCATGGCTGCTAGGCTGTGCTGCCAACTGGATCCTGCGCGGGACGTCCTTTGTTTACGTCCCGTCGGCGCTGAATCCCGCGGACGACCCCTCCCGGGGCCGCTTGGGGCTCTACCGCCCGCTTCTCCGCCTGTTCTACCGACCGACCACGGGGCGTACCTCTCTTTACGCGGACTCCCCGTCTGTGCCTTCTCATCTGCCGGACCGTGTGCACTTCGCTTCACCTCTGCACGTCGCATGGAGACCACCGTGAACGCCCACCGGAACTTGCCCAAGGTCTTGCATAAGAGGACTCTTGGACTTTCAGCAATGTCAACGACCGACCTTGAGGCATACTTCAAAGACTGTGTGTTTACTGAGTGGGAGGAGTTGGGGGAGGAGATTAGGTTAAAGGTCTTTGTACTAGGAGGCTGTAGGCATAAATTGGTGTGTTCACCAGCACCATGCAACTTTTTCACCTCTGCCTAATCATCTCATGTTCATGTCCTACTGTTCAAGCCTCCAAGCTGTGCCTTGGGTGGCTTTGGGGCATGGACATTGACACCTATAAAGAATTTGGAGCTTCTGTGGAGTTACTCTCTTTTTTGCCTTCTGACTTCTTTCCTTCTGTTCGAGATCTCCTCGACACCGCCTCTGCTCTGTATCGGGAGGCCTTAGAGTCTCCGGAACATTGTTCACCTCACCATACGGCACTCAGGCAAGCTATTGTGTGTTGGGGTGAGTTGATGAATCTAGCCACCTGGGTGGGAAGTAATTTGGAAGATCCAGCATCCAGGGAATTAGTAGTCAGCTATGTCAACGTTAATATGGGCCTAAAAATCAGACAACTATTGTGGTTTCACATTTCCTGTCTTACTTTTGGGAGAGAAACTGTTCTTGAATATTTGGTGTCTTTTGGAGTGTGGATTCGCACTCCTCCTGCATATAGACCACCAAATGCCCCTATCTTATCAACACTTCCGGAAACTACTGTTGTTAGACGAAGAGGCAGGTCCCCTAGAAGAAGAACTCCCTCGCCTCGCAGACGAAGGTCTCAATCGCCGCGTCGCAGAAGATCTCAATCTCGGGAATCTCAATGTTAGTATTCCTTGGACACATAAGGTGGGAAACTTTACGGGGCTTTATTCTTCTACGGTACCTTGCTTTAATCCTAAATGGCAAACTCCTTCTTTTCCTGACATTCATTTGCAGGAGGACATTGTTGATAGATGTAAGCAATTTGTGGGGCCCCTTACAGTAAATGAAAACAGGAGACTAAAATTAATTATGCCTGCTAGGTTTTATCCCAATGTTACTAAATATTTGCCCTTAGATAAAGGGATCAAACCTTATTATCCAGAGCATGTAGTTAATCATTACTTCCAGACGAGACATTATTTACACACTCTTTGGAAGGCGGGTATCTTATATAAAAGAGAGTCCACACGTAGCGCCTCATTTTGCGGGTCACCATATTCTTGGGAACAAGATCTACAGCATGGGAGGTTGGTCTTCCAAACCTCGAAAAGGCATGGGGACAAATCTTTCTGTCCCCAATCCCCTGGGATTCTTCCCCGATCATCAGTTGGACCCTGCATTCAAAGCCAACTCAGAAAATCCAGATTGGGACCTCAACCCGCACAAGGACAACTGGCCGGACGCCAACAAGGTGGGAGTGGGAGCATTCGGGCCAGGGTTCATCCCTCCCCATGGGGGACTGTTGGGGTGGAGCCCTCAGGCTCAGGGCATACTCACATCTGTGCCAGCAGCTCCTCCTCCTGCCTCCACCAATCGGCAGTCAGGAAGGCAGCCTACTCCCTTATCTCCACCTCTAAGGGACACTCATCCTCAGGCCATACAGTGGAA

**c16:**

CTCCACCACTTTCCACCAAACTCTTCAAGATCCAAGAGTCAGGGCCCTGTACTTTCCTGCTGGTGGCTCCAGTTCAGGAACAGTGAGCCCTGCTCAGAATACTGTCTCTGCCATATCGTCAATCTTATCGAAGACTGGGGACCCTGTACCGAACATGGAGAACATCGCATCACGACTCCTACGACCCCTGCTCGTGTTACAGGCGGGGTTTTTCTTGTTGACAAAAATCCTCACAATACCACAGAGTCTAGACTCGTGGTGGACTTCTCTCAATTTTCTAGGGGGAACACCCGTGTGTCTTGGCCAAAATTCGCAGTCCCAAATCTCCAGTCACTCACCAACCTGTTGTCCTCCAATTTGTCCTGGTTATCGCTGGATGTGTCTGCGGCGTTTTATCATCTTCCTTTGCATCCTGCTGCTATGCCTCATCTTCTTGTTGGTTCTTCTGGACTATCAAGGTATGTTGCCCGTTTGTCCTCTAATTCCAGGATCATCAACAACCAGCACCGGACCATGCAAAACCTGCACGACTCCTGCTCAAGGAACCTCTATGTTTCCCTCATGTTGCTGTACAAAACCTACGGACGGAAACTGCACCTGTATTCCCATCCCATCATCTTGGGCTTTCGCAAAATACCTATGGGAGTGGGCCTCAGTCCGTTTCTCTTGGCTCAGTTTACTAGTGCCATTTGTTCAGTGGTTCGTAGGGCTTTCCCCCACTGTCTGGCTTTCAGTTATATGGATGATGTGGTATTGGGGGCCAAGTCTGTACAACATCTTGAGTCCCTTTATGCCGCTGTTACCAATTTTCTTTTGTCTTTGGGTATACATTTAAACCCTCACAAAACAAAAAGATGGGGATATTCCCTTAACTTCATGGGCTATGTAATTGGGTGTTGGGGCACATTGCCACAGGAACATATTGTACAAAAAATCAAAATGTGTTTTAGAAAACTTCCTGTAAACAGACCTATTGATTGGAAAGTATGTCAACGAATTGTGGGTCTTTTGGGGTTTGCCGCCCCTTTCACGCAATGTGGATATCCTGCTTTAATGCCTTTATATGCATGTATACAAGCAAAACAGGCTTTTACTTTCTCGCCAACTTACAAGGCCTTTCTGAGTAAACAGTATCTGAACCTTTACCCCGTTGCTCGGCAACGGTCTGGTCTGTGCCAAGTGTTTGCTGACGCAACCCCCACTGGTTGGGGCTTGGCCATAGGCCATCAGCGCATGCGTGGAACCTTTGTGTCTCCTCTGCCGATCCATACTGCGGAACTCCTAGCCGCTTGTTTTGCTCGCAGCAGGTCTGGGGCAAAACTCATCGGGACTGACAATTCTGTCGTGCTCTCCCGCAAGTATACATCGTTCCCATGGCTGCTAGGCTGTGCTGCCAACTGGATCCTGCGCGGGACGTCCTTTGTTTACGTCCCGTCGGCGCTGAATCCCGCGGACGACCCCTCCCGGGGCCGCTTGGGGCTCTACCGCCCGCTTCTCCGCCTGTTCTACCGACCGACCACGGGGCGTACCTCTCTTTACGCGGACTCCCCGTCTGTGCCTTCTCATCTGCCGGACCGTGTGCACTTCGCTTCACCTCTGCACGTCGCATGGAGACCACCGTGAACGCCCACCGGAACTTGCCCAAGGTCTTGCATAAGAGGACTCTTGGACTTTCAGCAATGTCAACGACCGACCTTGAGGCATACTTCAAAGACTGTGTGTTTACTGAGTGGGAGGAGTTGGGGGAGGAGATTAGGTTAAAGGTCTTTGTACTAGGAGGCTGTAGGCATAAATTGGTGTGTTCACCAGCACCATGCAACTTTTTCACCTCTGCCTAATCATCTCATGTTCATGTCCTACTGTTCAAGCCTCCAAGCTGTGCCTTGGGTGGCTTTGGGGCATGGACATTGACACCTATAAAGAATTTGGAGCTTCTGTGGAGTTACTCTCTTTTTTGCCTTCTGACTTCTTTCCTTCTGTTCGAGATCTCCTCGACACCGCCTCTGCTCTGTATCGGGAGGCCTTAGAGTCTCCGGAACATTGTTCACCTCACCATACGGCACTCAGGCAAGCTATTGTGTGTTGGGGTGAGTTGATGAATCTAGCCACCTGGGTGGGAAGTAATTTGGAAGATCCAGCATCCAGGGAATTAGTAGTCAGCTATGTCAACGTTAATATGGGCCTAAAAATCAGACAACTATTGTGGTTTCACATTTCCTGTCTTACTTTTGGGAGAGAAACTGTTCTTGAATATTTGGTGTCTTTTGGAGTGTGGATTCGCACTCCTCCTGCATATAGACCACCAAATGCCCCTATCTTATCAACACTTCCGGAAACTACTGTTGTTAGACGAAGAGGCAGGTCCCCTAGAAGAAGAACTCCCTCGCCTCGCAGACGAAGGTCTCAATCGCCGCGTCGCAGAAGATCTCAATCTCGGGAATCTCAATGTTAGTATTCCTTGGACACATAAGGTGGGAAACTTTACTGGGCTTTATTCTTCTACGGTACCTTTCTTTAATCCTAAATGGCAAACTCCTTCTTTTCCTGACATTCATTTGCAGGAGGACATTGTTGATAGATGTAAGCAATTTGTGGGGCCCCTTACAGTAAATGAAAACAGGAGACTAAAATTAATTATGCCTGCTAGGTTTTATCCCAATGTTACTAAATATTTGCCCTTAGATAAAGGGATCAAACCTTATTATCCAGAGCATGTAGTTAATCATTACTTCCAGACGAGACATTATTTACACACTCTTTGGAAGGCGGGTATCTTATATAAAAGAGAGTCCACACGTAGCGCCTCATTTTGCGGGTCACCATATTCTTGGGAACAAGATCTACAGCATGGGAGGTTGGTCTTCCAAACCTCGAAAAGGCATGGGGACAAATCTTTCTGTCCCCAATCCCCTGGGATTCTTCCCCGATCATCAGTTGGACCCTGCATTCAAAGCCAACTCAGAAAATCCAGATTGGGACCTCAACCCGCACAAGGACAACTGGCCGGACGCCAACAAGGTGGGAGTGGGAGCATTCGGGCCAGGGTTCATCCCTCCCCATGGGGGACTGTTGGGGTGGAGCCCTCAGGCTCAGGGCATACTCACATCTGTGCCAGCAGCTCCTCCTCCTGCCTCCACCAATCGGCAGTCAGGAAGGCAGCCTACTCCCTTATCTCCACCTCTAAGGGACACTCATCCTCAGGCCATACAGTGGAA

**c17:**

CTCCACCACTTTCCACCAAACTCTTCAAGATCCAAGAGTCAGGGCCCTGTACTTTCCTGCTGGTGGCTCCAGTTCAGGAACAGTGAGCCCTGCTCAGAATACTGTCTCTGCCATATCGTCAATCTTATCGAAGACTGGGGACCCTGTACCGAACATGGAGAACATCGCATCAGGACTCCTAGGACCCCTGCTCGTGTTACAGGCGGGGTTTTTCTTGTTGACAAAAATCCTCACAATACCACAGAGTCTAGACTCGTGGTGGACTTCTCTCAATTTTCTAGGGGGAACACCCGTGTGTCTTGGCCAAAATTCGCAGTCCCAAATCTCCAGTCACTCACCAACCTGTTGTCCTCCAATTTGTCCTGGTTATCGCTGGATGTGTCTGCGGCGTTTTATCATCTTCCTTTGCATCCTGCTGCTATGCCTCATCTTCTTGTTGGTTCTTCTGGACTATCAAGGTATGTTGCCCGTTTGTCCTCTAATTCCAGGATCATCAACAACCAGCACCGGACCATGCAAAACCTGCACGACTCCTGCTCAAGGAACCTCTATGTTTCCCTCATGTTGCTGTACAAAACCTACGGACGGAAACTGCACCTGTATTCCCATCCCATCATCTTGGGCTTTCGCAAAATACCTATGGGAGTGGGCCTCAGTCCGTTTCTCTTGGCTCAGTTTACTAGTGCCATTTGTTCAGTGGTTCGTAGGGCTTTCCCCCACTGTCTGGCTTTCAGTTATATGGATGATGTGGTATTGGGGGCCAAGTCTGTACAACATCTTGAGTCCCTTTATGCCGCTGTTACCAATTTTCTTTTGTCTTTGGGTATACATTTAAACCCTCACAAAACAAAAAGATGGGGATATTCCCTTAACTTCATGGGCTATGTAATTGGGTGTTGGGGCACATTGCCACAGGAACATATTGTACAAAAAATCAAAATGTGTTTTAGAAAACTTCCTGTAAACAGACCTATTGATTGGAAAGTATGTCAACGAATTGTGGGTCTTTTGGGGTTTGCCGCCCCTTTCACGCAATGTGGATATCCTGCTTTAATGCCTTTATATGCATGTATACAAGCAAAACAGGCTTTTACTTTCTCGCCAACTTACAAGGCCTTTCTGAGTAAACAGTATCTGAACCTTTACCCCGTTGCTCGGCAACGGTCTGGTCTGTGCCAAGTGTTTGCTGACGCAACCCCCACTGGTTGGGGCTTGGCCATAGGCCATCAGCGCATGCGTGGAACCTTTGTGTCTCCTCTGCCGATCCATACTGCGGAACTCCTAGCCGCTTGTTTTGCTCGCAGCAGGTCTGGGGCAAAACTCATCGGGACTGACAATTCTGTCGTGCTCTCCCGCAAGTATACATCGTTCCCATGGCTGCTAGGCTGTGCTGCCAACTGGATCCTGCGCGGGACGTCCTTTGTTTACGTCCCGTCGGCGCTGAATCCCGCGGACGACCCCTCCCGGGGCCGCTTGGGGCTCTACCGCCCGCTTCTCCGCCTGTTCTACCGACCGACCACGGGGCGTACCTCTCTTTACGCGGACTCCCCGTCTGTGCCTTCTCATCTGCCGGACCGTGTGCACTTCGCTTCACCTCTGCACGTCGCATGGAGACCACCGTGAACGCCCACCGGAACTTGCCCAAGGTCTTGCATAAGAGGACTCTTGGACTTTCAGCAATGTCAACGACCGACCTTGAGGCATACTTCAAAGACTGTGTGTTTACTGAGTGGGAGGAGTTGGGGGAGGAGATTAGGTTAAAGGTCTTTGTACTAGGAGGCTGTAGGCATAAATTGGTGTGTTCACCAGCACCATGCAACTTTTTCACCTCTGCCTAATCATCTCATGTTCATGTCCTACTGTTCAAGCCTCCAAGCTGTGCCTTGGGTGGCTTTGGGGCATGGACATTGACACCTATAAAGAATTTGGAGCTTCTGTGGAGTTACTCTCTTTTTTGCCTTCTGACTTCTTTCCTTCTGTTCGAGATCTCCTCGACACCGCCTCTGCTCTGTATCGGGAGGCCTTAGAGTCTCCGGAACATTGTTCACCTCACCATACGGCACTCAGGCAAGCTATTGTGTGTTGGGGTGAGTTGATGAATCTAGCCACCTGGGTGGGAAGTAATTTGGAAGATCCAGCATCCAGGGAATTAGTAGTCAGCTATGTCAACGTTAATATGGGCCTAAAAATCAGACAACTATTGTGGTTTCACATTTCCTGTCTTACTTTTGGGAGAGAAACTGTTCTTGAATATTTGGTGTCTTTTGGAGTGTGGATTCGCACTCCTCCTGCATATAGACCACCAAATGCCCCTATCTTATCAACACTTCCGGAAACTACTGTTGTTAGACGAAGAGGCAGGTCCCCTAGAAGAAGAACTCCCTCGCCTCGCAGACGAAGGTCTCAATCGCCGCGTCGCAGAAGATCTCAATCTCGGGAATCTCAATGTTAGTATTCCTTGGACACATAAGGTGGGAAACTTTACGGGGCTTTATTCTTCTACGGTACCTTGCTTTAATCCTAAATGGCAAACTCCTTCTTTTCCTGACATTCATTTGCAGGAGGACATTGTTGATAGATGTAAGCAATTTGTGGGGCCCCTTACAGTAAATGAAAACAGGAGACTAAAATTAATTATGCCTGCTAGGTTTTATCCCAATGTTACTAAATATTTGCCCTTAGATAAAGGGATCAAACCTTATTATCCAGAGCATGTAGTTAATCATTACTTCCAGACGAGACATTATTTACACACTCTTTGGAAGGCGGGTATCTTATATAAAAGAGAGTCCACACGTAGCGCCTCATTTTGCGGGTCACCATATTCTTGGGAACAAGATCTACAGCATGGGAGGTTGGTCTTCCAAACCTCGAAAAGGCATGGGGACAAATCTTTCTGTCCCCAATCCCCTGGGATTCTTCCCCGATCATCAGTTGGACCCTGCATTCAAAGCCAACTCAGAAAATCCAGATTGGGACCTCAACCCGCACAAGGACAACTGGCCGGACGCCAACAAGGTGGGAGTGGGAGCATTCGGGCCAGGGTTCATCCCTCCCCATGGGGGACTGTTGGGGTGGAGCCCTCAGGCTCAGGGCATACTCACATCTGTGCCAGCAGCTCCTCCTCCTGCCTCCACCAATCGGCAGTCAGGAAGGCAGCCTACTCCCTTATCTCCACCTCTAAGGGACACTCATCCTCAGGCCATACAGTGGAA

**c22:**

CTCCACCACTTTCCACCAAACTCTTCAAGATCCAAGAGTCAGGGCCCTGTACTTTCCTGCTGGTGGCTCCAGTTCAGGAACAGTGAGCCCTGCTCAGAATACTGTCTCTGCCATATCGTCAATCTTATCGAAGACTGGGGACCCTGTACCGAACATGGAGAACATCGCATCAGGACTCCTAGGACCCCTGCTCGTGTTACAGGCGGGGTTTTTCTTGTTGACAAAAATCCTCACAATACCACAGAGTCTAGACTCGTGGTGGACTTCTCTCAATTTTCTAGGGGGAACACCCGTGTGTCTTGGCCAAAATTCGCAGTCCCAAATCTCCAGTCACTCACCAACCTGTTGTCCTCCAATTTGTCCTGGTTATCGCTGGATGTGTCTGCGGCGTTTTATCATCTTCCTTTGCATCCTGCTGCTATGCCTCATCTTCTTGTTGGTTCTTCTGGACTATCAAGGTATGTTGCCCGTTTGTCCTCTAATTCCAGGATCATCAACAACCAGCACCGGACCATGCAAAACCTGCACGACTCCTGCTCAAGGAACCTCTATGTTTCCCTCATGTTGCTGTACAAAACCTACGGACGGAAACTGCACCTGTATTCCCATCCCATCATCTTGGGCTTTCGCAAAATACCTATGGGAGTGGGCCTCAGTCCGTTTCTCTTGGCTCAGTTTACTAGTGCCATTTGTTCAGTGGTTCGTAGGGCTTTCCCCCACTGTCTGGCTTTCAGTTATATGGATGATGTGGTATTGGGGGCCAAGTCTGTACAACATCTTGAGTCCCTTTATGCCGCTGTTACCAATTTTCTTTTGTCTTTGGGTATACATTTAAACCCTCACAAAACAAAAAGATGGGGATATTCCCTTAACTTCATGGGCTATGTAATTGGGTGTTGGGGCACATTGCCACAGGAACATATTGTACAAAAAATCAAAATGTGTTTTAGAAAACTTCCTGTAAACAGACCTATTGATTGGAAAGTATGTCAACGAATTGTGGGTCTTTTGGGGTTTGCCGCCCCTTTCACGCAATGTGGATATCCTGCTTTAATGCCTTTATATGCATGTATACAAGCAAAACAGGCTTTTACTTTCTCGCCAACTTACAAGGCCTTTCTGAGTAAACAGTATCTGAACCTTTACCCCGTTGCTCGGCAACGGTCTGGTCTGTGCCAAGTGTTTGCTGACGCAACCCCCACTGGTTGGGGCTTGGCCATAGGCCATCAGCGCATGCGTGGAACCTTTGTGTCTCCTCTGCCGATCCATACTGCGGAACTCCTAGCCGCTTGTTTTGCTCGCAGCAGGTCTGGGGCAAAACTCATCGGGACTGACAATTCTGTCGTGCTCTCCCGCAAGTATACATCGTTCCCATGGCTGCTAGGCTGTGCTGCCAACTGGATCCTGCGCGGGACGTCCTTTGTTTACGTCCCGTCGGCGCTGAATCCCGCGGACGACCCCTCCCGGGGCCGCTTGGGGCTCTACCGCCCGCTTCTCCGCCTGTTCTACCGACCGACCACGGGGCGTACCTCTCTTTACGCGGACTCCCCGTCTGTGCCTTCTCATCTGCCGGACCGTGTGCACTTCGCTTCACCTCTGCACGTCGCATGGAGACCACCGTGAACGCCCACCGGAACTTGCCCAAGGTCTTGCATAAGAGGACTCTTGGACTTTCAGCAATGTCAACGACCGACCTTGAGGCATACTTCAAAGACTGTGTGTTTACTGAGTGGGAGGAGTTGGGGGAGGAGATTAGGTTAAAGGTCTTTGTACTAGGAGGCTGTAGGCATAAATTGGTGTGTTCACCAGCACCATGCAACTTTTTCACCTCTGCCTAATCATCTCATGTTCATGTCCTACTGTTCAAGCCTCCAAGCTGTGCCTTGGGTGGCTTTGGGGCATGGACATTGACACCTATAAAGAATTTGGAGCTTCTGTGGAGTTACTCTCTTTTTTGCCTTCTGACTTCTTTCCTTCTGTTCGAGATCTCCTCGACACCGCCTCTGCTCTGTATCGGGAGGCCTTAGAGTCTCCGGAACATTGTTCACCTCACCATACGGCACTCAGGCAAGCTATTGTGTGTTGGGGTGAGTTGATGAATCTAGCCACCTGGGTGGGAAGTAATTTGGAAGATCCAGCATCCAGGGAATTAGTAGTCAGCTATGTCAACGTTAATATGGGCCTAAAAATCAGACAACTATTGTGGTTTCACATTTCCTGTCTTACTTTTGGGAGAGAAACTGTTCTTGAATATTTGGTGTCTTTTGGAGTGTGGATTCGCACTCCTCCTGCATATAGACCACCAAATGCCCCTATCTTATCAACACTTCCGGAAACTACTGTTGTTAGACGAAGAGGCAGGTCCCCTAGAAGAAGAACTCCCTCGCCTCGCAGACGAAGGTCTCAATCGCCGCGTCGCAGAAGATCTCAATCTCGGGAATCTCAATGTTAGTATTCCTTGGACACATAAGGTGGGAAACTTTACGGGGCTTTATTCTTCTACGGTACCTTGCTTTAATCCTAAATGGCAAACTCCTTCTTTTCCTGACATTCATTTGCAGGAGGACATTGTTGATAGATGTAAGCAATTTGTGGGGCCCCTTACAGTAAATGAAAACAGGAGACTAAAATTAATTATGCCTGCTAGGTTTTATCCCAATGTTACTAAATATTTGCCCTTAGATAAAGGGATCAAACCTTATTATCCAGAGCATGTAGTTAATCATTACTTCCAGACGAGACATTATTTACACACTCTTTGGAAGGCGGGTATCTTATATAAAAGAGAGTCCACACGTAGCGCCTCATTTTGCGGGTCACCATATTCTTGGGAACAAGATCTACAGCATGGGAGGTTGGTCTTCCAAACCTCGAAAAGGCATGGGGACAAATCTTTCTGTCCCCAATCCCCTGGGATTCTTCCCCGATCATCAGTTGGACCCTGCATTCAAAGCCAACTCAGAAAATCCAGATTGGGACCTCAACCCGCACAAGGACAACTGGCCGGACGCCAACAAGGTGGGAGTGGGAGCATTCGGGCCAGGGTTCATCCCTCCCCATGGGGGACTGTTGGGGTGGAGCCCTCAGGCTCAGGGCATACTCACATCTGTGCCAGCAGCTCCTCCTCCTGCCTCCACCAATCGGCAGTCAGGAAGGCAGCCTACTCCCTTATCTCCACCTCTAAGGGACACTCATCCTCAGGCCATACAGTGGAA

**Genotype C:**

**c14:**

CTCCACAACATTCCACCAAGCTCTGCTAGATCCCAGAGTGAGGGGCCTATATTTTCCTGCTGGTGGCTCCAGTTCCGGAACAGTAAACCCTGTTCCGACTACTGCCTCACCCATATCGTCAATCTTCTCGAGGACTGGGGACCCTGCACCGAACATGGAGAACACAACATCAGGATTCCTAGGACCCCTGCTCGTGTTACAGGCGGGGTTTTTCTTGTTGACAAAAATCCTCACAATACCACAGAGTCTAGACTCGTGGTGGACTTCTCTCAATTTTCTAGGGGGAGCACCCAAGTGTCCTGGCCAAAATTCGCAGTCCCCAACCTCCAATCACTTACCAACCTCTTGTCCTCCAATTTGTCCTGGCTATCGCTGGATGTGTCTGCGGCGTTTTATCATATTCCTCTTCATCCTGCTGCTATGCCTCATCTTCTTGTTGGTTCTTCTGGACTGCCAAGGTATGTTGCCCGTTTGTCCTCTACTTCCAGGAACATCAACCACCAGCACGGGGCCATGCAAGACCTGCACGACTCCTGCTCAAGGAACCTCTATGTTTCCCTCTTGTTGCTGTACAAAACCTTCGGACGGAAACTGCACTTGTATTCCCATCCCATCATCTTGGGCTTTCGCAAGATTCCTATGGGAGTGGGCCTCAGTCCGTTTCTCCTGGCTCAGTTTACTAGTGCCATTTGTTCAGTGGTTCGTAGGGCTTTCCCCCACTGTTTGGCTTTCAGTTATATGGATGATGTGGTATTGGGGGCCAAGTCTGTACAACATCTTGAGTCCCTTTTTACCTCTATTACCAATTTTCTTCTGTCTTTGGGTATACATTTAAACCCTAATAAAACCAAACGTTGGGGCTACTCCCTTAACTTTATGGGATATGTAATTGGAAGTTGGGGTACTTTACCGCAGGAACATATTGTACTAAAACTCAAGCAATGTTTTCGAAAATTGCCTGTCAATAGACCTATTGATTGGAAAGTATGTCAAAGAATTGTGGGTCTTTTGGGCTTTGCTGCCCCTTTTACACAATGTGGCTATCCTGCCTTGATGCCTTTATATGCATGTATACAATCTAAGCAGGCTTTCACTTTCTCGCCAACTTACAAGGCCTTTCTGTGTAAACAATATATGCACCTTTACCCCGTTGCCCGGCAACGGTCAGGCCTCTGCCAAGTGTTTGCTGACGCAACCCCCACTGGATGGGGCTTGGCCATAGGCCATCAGCGCATGCGTGGAACCTTTGTGGCTCCTCTGCCGATCCATACTGCGGAACTCCTAGCCGCTTGTTTTGCTCGCAGCCGGTCTGGAGCGAAACTTATCGGAACCGACAACTCTGTTGTCCTCTCTCGGAAATACACCTCCTTTCCATGGCTGCTAGGATGTGCTGCCAACTGGATCCTGCGCGGGACGTCCTTTGTCTACGTCCCGTCGGCGCTGAATCCCGCGGACGACCCGTCTCGGGGCCGTTTGGGACTCTACCGTCCCCTTCTTCGTCTGCCGTTCCGGCCAACCACGGGGCGCACCTCTCTTTACGCGGTCTCCCCGTCTGTGCCTTCTCATCTGCCGGACCGTGTGCACTTCGCTTCACCTCTGCACGTCGCATGGAAACCACCGTGAACGCCCACCAGGTCTTGCCCAAGGTCTTATATAAGAGGACTCTTGGACTCTCAGCAATGTCAACGACCGACCTTGAGGCATACTTCAAAGACTGTGTGTTTAAAGACTGGGAGGAGTTGGGGGAGGAGATTAGGTTAATGATCTTTGTACTAGGAGGCTGTAGGCATAAATTGGTGTGTTCACCAGCACCATGCAACTTTTTCACCTCTGCCTAATCATCTCATGTTCATGTCCTACTGTTCAAGCCTCCAAGCTGTGCCTTGGGTGGCTTTGGGGCATGGACATTGACCCGTATAAAGAATTTGGAGCATCTGTGGAGTTACTCTCTTTTTTGCCTTCTGACTTCTTTCCTTCTATTCGAGATCTCCTCGACACCGCCTCTGCTCTGTATCGGGAGGCCTTGGAGTCTCCGGAACATTGTTCACCTCACCATACAGCACTCAGGCAAGCTATTCTGTGTTGGGGTGAGTTGATGAATCTGGCCACCTGGGTGGGAAGTAATTTGGAAGACCCAGCATCCAGGGAATTAGTAGTCGGCTATGTCAACGTTAATATGGGCCTAAAACTCAGACAACTATTGTGGTTTCACATTTCCTGTCTTACTTTTGGAAGAGAAACTGTTCTTGAGTATTTGGTATCTTTTGGAGTGTGGATTCGCACTCCTACTGCTTACAGACCACCAAATGCCCCTATCTTATCAACACTTCCGGAAACTACTGTTGTTAGACGACGAGGCAGGTCCCCTAGAAGAAGAACTCCCTCGCCTCGCAGACGAAGGTCTCAATCGCCGCGTCGCAGAAGATCTCAATCTCGGGAATCTCAATGTTAGTATCCCTTGGACTCATAAGGTGGGAAACTTTACTGGGCTTTATTCTTCTACTGTACCTGTCTTTAATCCTGGGTGGCAAACTCCCTCCTTTCCTCACATTCATTTACGGGAGGACATTATTGATAGATGTCAACAATATGTAGGCCCTCTTACAATTAATGAAAAAAGGAGATTAAAATTAATTATGCCTGCTAGGTTTTATCCTAACCTTACCAAATATTTGCCCTTGGACAAAGGCATTAAACCGTATTATCCGGAACATGCAGTTAATCATTACTTCAAAACCAGGCATTATTTACATACTCTGTGGAAGGCTGGCATTCTATATAAGAGAGAAACTACACGTAGCGCCTCATTCTGTGGGTCACCATATTCTTGGGAACAAGAGCTACGGCATGGGAGGTCCGTCTTCCAAACCTCGACGAGGCATGGGGACGAATCTTGCGGTTCCCAATCCTCTGGGATTCTTTCCCGATCACCAGTTGGACCTTGCGTTCAGAGCCAACTCAAACAATCCAGATTGGGACTTCAACCCCAACAAGGATCCCTGGCCAGAGGCAAATCAGGTGGGAGCGGGAGCATTCGGGCCAGGGTTCACCCCACCACACGGCGGTCTTCTGGGGTGGAGCCCTCAGGCTCAGGGCATATTGACAACAGTGCCAGCAGCACCTCCTCCTGCCTCCACCAATCGGCAGGCAGGAAGAAAGCCTACTCCCATCTCTCCACCTCTAAGAGACAGTCATCCTCAGGCCGTGCAGTGGAA

**c15:**

CTCCACAACATTCCACCAAGCTCTGCTAGATCCCAGAGTGAGGGGCCTATATTTTCCTGCTGGTGGCTCCAGTTCCGGAACAGTAAACCCTGTTCCGACTACTGCCTCACCCATATCGTCAATCTTCTCGAGGACTGGGGACCCTGCACCGAACATGGAGAACACAACATCAGGATTCCTAGGACCCCTGCTCGTGTTACAGGCGGGGTTTTTCTTGTTGACAAAAATCCTCACAATACCACAGAGTCTAGACTCGTGGTGGACTTCTCTCAATTTTCTAGGGGGAGCACCCAAGTGTCCTGGCCAAAATTCGCAGTCCCCAACCTCCAATCACTTACCAACCTCTTGTCCTCCAATTTGTCCTGGCTATCGCTGGATGTGTCTGCGGCGTTTTATCATATTCCTCTTCATCCTGCTGCTATGCCTCATCTTCTTGTTGGTTCTTCTGGACTGCCAAGGTATGTTGCCCGTTTGTCCTCTACTTCCAGGAACATCAACCACCAGCACGGGGCCATGCAAGACCTGCACGACTCCTGCTCAAGGAACCTCTATGTTTCCCTCTTGTTGCTGTACAAAACCTTCGGACGGAAACTGCACTTGTATTCCCATCCCATCATCTTGGGCTTTCGCAAGATTCCTATGGGAGTGGGCCTCAGTCCGTTTCTCCTGGCTCAGTTTACTAGTGCCATTTGTTCAGTGGTTCGTAGGGCTTTCCCCCACTGTTTGGCTTTCAGTTATATGGATGATGTGGTATTGGGGGCCAAGTCTGTACAACATCTTGAGTCCCTTTTTACCTCTATTACCAATTTTCTTCTGTCTTTGGGTATACATTTAAACCCTAATAAAACCAAACGTTGGGGCTACTCCCTTAACTTTATGGGATATGTAATTGGAAGTTGGGGTACTTTACCGCAGGAACATATTGTACTAAAACTCAAGCAATGTTTTCGAAAATTGCCTGTCAATAGACCTATTGATTGGAAAGTATGTCAAAGAATTGTGGGTCTTTTGGGCTTTGCTGCCCCTTTTACACAATGTGGCTATCCTGCCTTGATGCCTTTATATGCATGTATACAATCTAAGCAGGCTTTCACTTTCTCGCCAACTTACAAGGCCTTTCTGTGTAAACAATATATGCACCTTTACCCCGTTGCCCGGCAACGGTCAGGCCTCTGCCAAGTGTTTGCTGACGCAACCCCCACTGGATGGGGCTTGGCCATAGGCCATCAGCGCATGCGTGGAACCTTTGTGGCTCCTCTGCCGATCCATACTGCGGAACTCCTAGCCGCTTGTTTTGCTCGCAGCCGGTCTGGAGCGAAACTTATCGGAACCGACAACTCTGTTGTCCTCTCTCGGAAATACACCTCCTTTCCATGGCTGCTAGGATGTGCTGCCAACTGGATCCTGCGCGGGACGTCCTTTGTCTACGTCCCGTCGGCGCTGAATCCCGCGGACGACCCGTCTCGGGGCCGTTTGGGACTCTACCGTCCCCTTCTTCGTCTGCCGTTCCGGCCAACCACGGGGCGCACCTCTCTTTACGCGGTCTCCCCGTCTGTGCCTTCTCATCTGCCGGACCGTGTGCACTTCGCTTCACCTCTGCACGTCGCATGGAAACCACCGTGAACGCCCACCAGGTCTTGCCCAAGGTCTTATATAAGAGGACTCTTGGACTCTCAGCAATGTCAACGACCGACCTTGAGGCATACTTCAAAGACTGTGTGTTTAAAGACTGGGAGGAGTTGGGGGAGGAGATTAGGTTAATGATCTTTGTACTAGGAGGCTGTAGGCATAAATTGGTGTGTTCACCAGCACCATGCAACTTTTTCACCTCTGCCTAATCATCTCATGTTCATGTCCTACTGTTCAAGCCTCCAAGCTGTGCCTTGGGTGGCTTTGGGGCATGGACATTGACCCGTATAAAGAATTTGGAGCATCTGTGGAGTTACTCTCTTTTTTGCCTTCTGACTTCTTTCCTTCTATTCGAGATCTCCTCGACACCGCCTCTGCTCTGTATCGGGAGGCCTTGGAGTCTCCGGAACATTGTTCACCTCACCATACAGCACTCAGGCAAGCTATTCTGTGTTGGGGTGAGTTGATGAATCTGGCCACCTGGGTGGGAAGTAATTTGGAAGACCCAGCATCCAGGGAATTAGTAGTCGGCTATGTCAACGTTAATATGGGCCTAAAACTCAGACAACTATTGTGGTTTCACATTTCCTGTCTTACTTTTGGAAGAGAAACTGTTCTTGAGTATTTGGTATCTTTTGGAGTGTGGATTCGCACTCCTACTGCTTACAGACCACCAAATGCCCCTATCTTATCAACACTTCCGGAAACTACTGTTGTTAGACGACGAGGCAGGTCCCCTAGAAGAAGAACTCCCTCGCCTCGCAGACGAAGGTCTCAATCGCCGCGTCGCAGAAGATCTCAATCTCGGGAATCTCAATGTTAGTATCCCTTGGACTCATAAGGTGGGAAACTTTACTGGGCTTTATTCTTCTACTGTACCTGTCTTTAATCCTGGGTGGCAAACTCCCTCCTTTCCTCACATTCATTTACGGGAGGACATTATTGATAGATGTCAACAATATGTAGGCCCTCTTACAATTAATGAAAAAAGGAGATTAAAATTAATTATGCCTGCTAGGTTTTATCCTAACCTTACCAAATATTTGCCCTTGGACAAAGGCATTAAACCGTATTATCCGGAACATGCAGTTAATCATTACTTCAAAACCAGGCATTATTTACATACTCTGTGGAAGGCTGGCATTCTATATAAGAGAGAAACTACACGTAGCGCCTCATTCTGTGGGTCACCATATTCTTGGGAACAAGAGCTACGGCATGGGAGGTCCGTCTTCCAAACCTCGACGAGGCATGGGGACGAATCTTGCGGTTCCCAATCCTCTGGGATTCTTTCCCGATCACCAGTTGGACCTTGCGTTCAGAGCCAACTCAAACAATCCAGATTGGGACTTCAACCCCAACAAGGATCCCTGGCCAGAGGCAAATCAGGTGGGAGCGGGAGCATTCGGGCCAGGGTTCACCCCACCACACGGCGGTCTTCTGGGGTGGAGCCCTCAGGCTCAGGGCATATTGACAACAGTGCCAGCAGCACCTCCTCCTGCCTCCACCAATCGGCAGGCAGGAAGAAAGCCTACTCCCATCTCTCCACCTCTAAGAGACAGTCATCCTCAGGCCGTGCAGTGGAA

**c20:**

CTCCACAACATTCCACCAAGCTCTGCTAGATCCCAGAGTGAGGGGCCTATATTTTCCTGCTGGTGGCTCCAGTTCCGGAACAGTAAACCCTGTTCCGACTACTGCCTCACCCATATCGTCAATCTTCTCGAGGACTGGGGACCCTGCACCGAACATGGAGAACACAACATCAGGATTCCTAGGACCCCTGCTCGTGTTACAGGCGGGGTTTTTCTTGTTGACAAAAATCCTCACAATACCACAGAGTCTAGACTCGTGGTGGACTTCTCTCAATTTTCTAGGGGGAGCACCCAAGTGTCCTGGCCAAAATTCGCAGTCCCCAACCTCCAATCACTTACCAACCTCTTGTCCTCCAATTTGTCCTGGCTATCGCTGGATGTGTCTGCGGCGTTTTATCATATTCCTCTTCATCCTGCTGCTATGCCTCATCTTCTTGTTGGTTCTTCTGGACTGCCAAGGTATGTTGCCCGTTTGTCCTCTACTTCCAGGAACATCAACCACCAGCACGGGGCCATGCAAGACCTGCACGACTCCTGCTCAAGGAACCTCTATGTTTCCCTCTTGTTGCTGTACAAAACCTTCGGACGGAAACTGCACTTGTATTCCCATCCCATCATCTTGGGCTTTCGCAAGATTCCTATGGGAGTGGGCCTCAGTCCGTTTCTCCTGGCTCAGTTTACTAGTGCCATTTGTTCAGTGGTTCGTAGGGCTCTCCCCCACTGTTTGGCTTTCAGTTATATGGATGATGTGGTATTGGGGGCCAAGTCTGTACAACATCTTGAGTCCCTTTTTACCTCTATTACCAATTTTCTTCTGTCTTTGGGTATACATTTAAACCCTAATAAAACCAAACGTTGGGGCTACTCCCTTAACTTTATGGGATATGTAATTGGAAGTTGGGGTACTTTACCGCAGGAACATATTGTACTAAAACTCAAGCAATGTTTTCGAAAATTGCCTGTCAATAGACCTATTGATTGGAAAGTATGTCAAAGAATTGTGGGTCTTTTGGGCTTTGCTGCCCCTTTTACACAATGTGGCTATCCTGCCTTGATGCCTTTATATGCATGTATACAATCTAAGCAGGCTTTCACTTTCTCGCCAACTTACAAGGCCTTTCTGTGTAAACAATATATGCACCTTTACCCCGTTGCCCGGCAACGGTCAGGCCTCTGCCAAGTGTTTGCTGACGCAACCCCCACTGGATGGGGCTTGGCCATAGGCCATCAGCGCATGCGTGGAACCTTTGTGGCTCCTCTGCCGATCCATACTGCGGAACTCCTAGCCGCTTGTTTTGCTCGCAGCCGGTCTGGAGCGAAACTTATCGGAACCGACAACTCTGTTGTCCTCTCTCGGAAATACACCTCCTTTCCATGGCTGCTAGGATGTGCTGCCAACTGGATCCTGCGCGGGACGTCCTTTGTCTACGTCCCGTCGGCGCTGAATCCCGCGGACGACCCGTCTCGGGGCCGTTTGGGACTCTACCGTCCCCTTCTTCGTCTGCCGTTCCGGCCAACCACGGGGCGCACCTCTCTTTACGCGGTCTCCCCGTCTGTGCCTTCTCATCTGCCGGACCGTGTGCACTTCGCTTCACCTCTGCACGTCGCATGGAAACCACCGTGAACGCCCACCAGGTCTTGCCCAAGGTCTTATATAAGAGGACTCTTGGACTCTCAGCAATGTCAACGACCGACCTTGAGGCATACTTCAAAGACTGTGTGTTTAAAGACTGGGAGGAGTTGGGGGAGGAGATTAGGTTAATGATCTTTGTACTAGGAGGCTGTAGGCATAAATTGGTGTGTTCACCAGCACCATGCAACTTTTTCACCTCTGCCTAATCATCTCATGTTCATGTCCTACTGTTCAAGCCTCCAAGCTGTGCCTTGGGTGGCTTTGGGGCATGGACATTGACCCGTATAAAGAATTTGGAGCATCTGTGGAGTTACTCTCTTTTTTGCCTTCTGACTTCTTTCCTTCTATTCGAGATCTCCTCGACACCGCCTCTGCTCTGTATCGGGAGGCCTTGGAGTCTCCGGAACATTGTTCACCTCACCATACAGCACTCAGGCAAGCTATTCTGTGTTGGGGTGAGTTGATGAATCTGGCCACCTGGGTGGGAAGTAATTTGGAAGACCCAGCATCCAGGGAATTAGTAGTCGGCTATGTCAACGTTAATATGGGCCTAAAACTCAGACAACTATTGTGGTTTCACATTTCCTGTCTTACTTTTGGAAGAGAAACTGTTCTTGAGTATTTGGTATCTTTTGGAGTGTGGATTCGCACTCCTACTGCTTACAGACCACCAAATGCCCCTATCTTATCAACACTTCCGGAAACTACTGTTGTTAGACGACGAGGCAGGTCCCCTAGAAGAAGAACTCCCTCGCCTCGCAGACGAAGGTCTCAATCGCCGCGTCGCAGAAGATCTCAATCTCGGGAATCTCAATGTTAGTATCCCTTGGACTCATAAGGTGGGAAACTTTACTGGGCTTTATTCTTCTACTGTACCTGTCTTTAATCCTGGGTGGCAAACTCCCTCCTTTCCTCACATTCATTTACGGGAGGACATTATTGATAGATGTCAACAATATGTAGGCCCTCTTACAATTAATGAAAAAAGGAGATTAAAATTAATTATGCCTGCTAGGTTTTATCCTAACCTTACCAAATATTTGCCCTTGGACAAAGGCATTAAACCGTATTATCCGGAACATGCAGTTAATCATTACTTCAAAACCAGGCATTATTTACATACTCTGTGGAAGGCTGGCATTCTATATAAGAGAGAAACTACACGTAGCGCCTCATTCTGTGGGTCACCATATTCTTGGGAACAAGAGCTACGGCATGGGAGGTCCGTCTTCCAAACCTCGACGAGGCATGGGGACGAATCTTGCGGTTCCCAATCCTCTGGGATTCTTTCCCGATCACCAGTTGGACCTTGCGTTCAGAGCCAACTCAAACAATCCAGATTGGGACTTCAACCCCAACAAGGATCCCTGGCCAGAGGCAAATCAGGTGGGAGCGGGAGCATTCGGGCCAGGGTTCACCCCACCACACGGCGGTCTTCTGGGGTGGAGCCCTCAGGCTCAGGGCATATTGACAACAGTGCCAGCAGCACCTCCTCCTGCCTCCACCAATCGGCAGGCAGGAAGAAAGCCTACTCCCATCTCTCCACCTCTAAGAGACAGTCATCCTCAGGCCGTGCAGTGGAA

**c21:**

CTCCACAACATTCCACCAAGCTCTGCTAGATCCCAGAGTGAGGGGCCTATATTTTCCTGCTGGTGGCTCCAGTTCCGGAACAGTAAACCCTGTTCCGACTACTGCCTCACCCATATCGTCAATCTTCTCGAGGACTGGGGACCCTGCACCGAACATGGAGAACACAACATCAGGATTCCTAGGACCCCTGCTCGTGTTACAGGCGGGGTTTTTCTTGTTGACAAAAATCCTCACAATACCACAGAGTCTAGACTCGTGGTGGACTTCTCTCAATTTTCTAGGGGGAGCACCCAAGTGTCCTGGCCAAAATTCGCAGTCCCCAACCTCCAATCACTTACCAACCTCTTGTCCTCCAATTTGTCCTGGCTATCGCTGGATGTGTCTGCGGCGTTTTATCATATTCCTCTTCATCCTGCTGCTATGCCTCATCTTCTTGTTGGTTCTTCTGGACTGCCAAGGTATGTTGCCCGTTTGTCCTCTACTTCCAGGAACATCAACCACCAGCACGGGGCCATGCAAGACCTGCACGACTCCTGCTCAAGGAACCTCTATGTTTCCCTCTTGTTGCTGTACAAAACCTTCGGACGGAAACTGCACTTGTATTCCCATCCCATCATCTTGGGCTTTCGCAAGATTCCTATGGGAGTGGGCCTCAGTCCGTTTCTCCTGGCTCAGTTTACTAGTGCCATTTGTTCAGTGGTTCGTAGGGCTTTCCCCCACTGTTTGGCTTTCAGTTATATGGATGATGTGGTATTGGGGGCCAAGTCTGTACAACATCTTGAGTCCCTTTTTACCTCTATTACCAATTTTCTTCTGTCTTTGGGTATACATTTAAACCCTAATAAAACCAAACGTTGGGGCTACTCCCTTAACTTTATGGGATATGTAATTGGAAGTTGGGGTACTTTACCGCAGGAACATATTGTACTAAAACTCAAGCAATGTTTTCGAAAATTGCCTGTCAATAGACCTATTGATTGGAAAGTATGTCAAAGAATTGTGGGTCTTTTGGGCTTTGCTGCCCCTTTTACACAATGTGGCTATCCTGCCTTGATGCCTTTATATGCATGTATACAATCTAAGCAGGCTTTCACTTTCTCGCCAACTTACAAGGCCTTTCTGTGTAAACAATATATGCACCTTTACCCCGTTGCCCGGCAACGGTCAGGCCTCTGCCAAGTGTTTGCTGACGCAACCCCCACTGGATGGGGCTTGGCCATAGGCCATCAGCGCATGCGTGGAACCTTTGTGGCTCCTCTGCCGATCCATACTGCGGAACTCCTAGCCGCTTGTTTTGCTCGCAGCCGGTCTGGAGCGAAACTTATCGGAACCGACAACTCTGTTGTCCTCTCTCGGAAATACACCTCCTTTCCATGGCTGCTAGGATGTGCTGCCAACTGGATCCTGCGCGGGACGTCCTTTGTCTACGTCCCGTCGGCGCTGAATCCCGCGGACGACCCGTCTCGGGGCCGTTTGGGACTCTACCGTCCCCTTCTTCGTCTGCCGTTCCGGCCAACCACGGGGCGCACCTCTCTTTACGCGGTCTCCCCGTCTGTGCCTTCTCATCTGCCGGACCGTGTGCACTTCGCTTCACCTCTGCACGTCGCATGGAAACCACCGTGAACGCCCACCAGGTCTTGCCCAAGGTCTTATATAAGAGGACTCTTGGACTCTCAGCAATGTCAACGACCGACCTTGAGGCATACTTCAAAGACTGTGTGTTTAAAGACTGGGAGGAGTTGGGGGAGGAGATTAGGTTAATGATCTTTGTACTAGGAGGCTGTAGGCATAAATTGGTGTGTTCACCAGCACCATGCAACTTTTTCACCTCTGCCTAATCATCTCATGTTCATGTCCTACTGTTCAAGCCTCCAAGCTGTGCCTTGGGTGGCTTTGGGGCATGGACATTGACCCGTATAAAGAATTTGGAGCATCTGTGGAGTTACTCTCTTTTTTGCCTTCTGACTTCTTTCCTTCTATTCGAGATCTCCTCGACACCGCCTCTGCTCTGTATCGGGAGGCCTTGGAGTCTCCGGAACATTGTTCACCTCACCATACAGCACTCAGGCAAGCTATTCTGTGTTGGGGTGAGTTGATGAATCTGGCCACCTGGGTGGGAAGTAATTTGGAAGACCCAGCATCCAGGGAATTAGTAGTCGGCTATGTCAACGTTAATATGGGCCTAAAACTCAGACAACTATTGTGGTTTCACATTTCCTGTCTTACTTTTGGAAGAGAAACTGTTCTTGAGTATTTGGTATCTTTTGGAGTGTGGATTCGCACTCCTACTGCTTACAGACCACCAAATGCCCCTATCTTATCAACACTTCCGGAAACTACTGTTGTTAGACGACGAGGCAGGTCCCCTAGAAGAAGAACTCCCTCGCCTCGCAGACGAAGGTCTCAATCGCCGCGTCGCAGAAGATCTCAATCTCGGGAATCTCAATGTTAGTATCCCTTGGACTCATAAGGTGGGAAACTTTACTGGGCTTTATTCTTCTACTGTACCTGTCTTTAATCCTGGGTGGCAAACTCCCTCCTTTCCTCACATTCATTTACGGGAGGACATTATTGATAGATGTCAACAATATGTAGGCCCTCTTACAATTAATGAAAAAAGGAGATTAAAATTAATTATGCCTGCTAGGTTTTATCCTAACCTTACCAAATATTTGCCCTTGGACAAAGGCATTAAACCGTATTATCCGGAACATGCAGTTAATCATTACTTCAAAACCAGGCATTATTTACATACTCTGTGGAAGGCTGGCATTCTATATAAGAGAGAAACTACACGTAGCGCCTCATTCTGTGGGTCACCATATTCTTGGGAACAAGAGCTACGGCATGGGAGGTCCGTCTTCCAAACCTCGACGAGGCATGGGGACGAATCTTGCGGTTCCCAATCCTCTGGGATTCTTTCCCGATCACCAGTTGGACCTTGCGTTCAGAGCCAACTCAAACAATCCAGATTGGGACTTCAACCCCAACAAGGATCCCTGGCCAGAGGCAAATCAGGTGGGAGCGGGAGCATTCGGGCCAGGGTTCACCCCACCACACGGCGGTCTTCTGGGGTGGAGCCCTCAGGCTCAGGGCATATTGACAACAGTGCCAGCAGCACCTCCTCCTGCCTCCACCAATCGGCAGGCAGGAAGAAAGCCTACTCCCATCTCTCCACCTCTAAGAGACAGTCATCCTCAGGCCGTGCAGTGGAA
